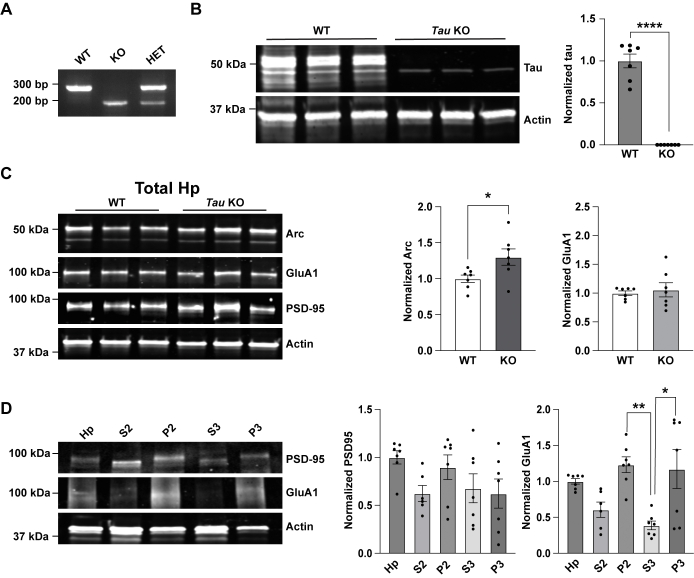# Tau regulates Arc stability in neuronal dendrites *via* a proteasome-sensitive but ubiquitin-independent pathway

**DOI:** 10.1016/j.jbc.2024.107237

**Published:** 2024-03-27

**Authors:** Dina W. Yakout, Ankit Shroff, Wei Wei, Vishrut Thaker, Zachary D. Allen, Mathew Sajish, Taras Y. Nazarko, Angela M. Mabb

**Affiliations:** 1 Neuroscience Institute, Georgia State University, Atlanta, Georgia, USA; 2 Department of Biology, Georgia State University, Atlanta, Georgia, USA; 3 Department of Drug Discovery and Biomedical Sciences, College of Pharmacy, University of South Carolina, Columbia, South Carolina, USA; 4 Center for Behavioral Neuroscience, Georgia State University, Atlanta, Georgia, USA

**Keywords:** arc, tau, AMPA receptors, proteasome, ubiquitination, Alzheimer’s disease

## Abstract

Tauopathies are neurodegenerative disorders characterized by the deposition of aggregates of the microtubule-associated protein tau, a main component of neurofibrillary tangles. Alzheimer’s disease (AD) is the most common type of tauopathy and dementia, with amyloid-beta pathology as an additional hallmark feature of the disease. Besides its role in stabilizing microtubules, tau is localized at postsynaptic sites and can regulate synaptic plasticity. The activity-regulated cytoskeleton-associated protein (Arc) is an immediate early gene that plays a key role in synaptic plasticity, learning, and memory. Arc has been implicated in AD pathogenesis and regulates the release of amyloid-beta. We found that decreased Arc levels correlate with AD status and disease severity. Importantly, Arc protein was upregulated in the hippocampus of *Tau* KO mice and dendrites of *Tau* KO primary hippocampal neurons. Overexpression of tau decreased Arc stability in an activity-dependent manner, exclusively in neuronal dendrites, which was coupled to an increase in the expression of dendritic and somatic surface GluA1-containing α-amino-3-hydroxy-5-methyl-4-isoxazolepropionic acid receptors. The tau-dependent decrease in Arc was found to be proteasome-sensitive, yet independent of Arc ubiquitination and required the endophilin-binding domain of Arc. Importantly, these effects on Arc stability and GluA1 localization were not observed in the commonly studied tau mutant, P301L. These observations provide a potential molecular basis for synaptic dysfunction mediated through the accumulation of tau in dendrites. Our findings confirm that Arc is misregulated in AD and further show a physiological role for tau in regulating Arc stability and AMPA receptor targeting.

Tauopathies are a diverse group of neurodegenerative disorders predominantly characterized by dementia or degeneration of the motor system (1). A hallmark of tauopathies is the accumulation of tau into insoluble aggregates and filaments which is a major component of neurofibrillary tangles (NFTs) in the brain (2, 3). Besides tau pathology, tauopathies may also involve other pathological changes such as amyloid deposition that is observed in Alzheimer’s disease (AD) and Down’s syndrome (1). In AD, the most common tauopathy, the progress of tau pathology, follows a stereotypical pattern in the brain that is highly correlated with the progress of cognitive impairment, which led Braak and Braak to base the staging of AD on the pattern of NFT deposition in the brain (4).

Tau is encoded by the *MAPT* gene located on chromosome 17 (5). Its C-terminal region contains 18-residue repeats, which together form the microtubule-binding domain (MTBD), which is linked to the N-terminal region through a proline-rich region (6). The *MAPT* gene consists of 16 exons, 11 of which are expressed in the central nervous system (7). In humans, six different isoforms of tau have been reported with differences in alternative mRNA splicing of exons *2*, *3*, and *10*. Alternative splicing of exons *2* and *3* yields 0, 1, or 2 N-terminal repeats (0N, 1N, and 2N isoforms) while alternative splicing of exon *10* leads to the presence or absence of the R2 domain, which is one of the four repeats that bind to microtubules (3R and 4R isoforms) (8). Tau also undergoes several posttranslational modifications including, but not limited to, phosphorylation (9), acetylation (10, 11), and ubiquitination (12). During early stages of development, tau is highly phosphorylated compared to the adult brain (13). In tauopathies, tau becomes hyperphosphorylated, which is thought to increase its propensity to form aggregates and reduce its affinity for microtubules (14). Additionally, it has been shown that some degree of tau accumulation and hyperphosphorylation occurs in normal aging (15, 16).

There are over 50 mutants of the *MAPT* gene that have been identified in several tauopathies (17). Some of these mutations can affect the alternative splicing of *tau* mRNA leading to overproduction of 3R or 4R isoforms and thus pathologically increasing tau and facilitating its aggregation (18). Other mutations, like the missense P301L mutation (found within the R2 region), increase tau phosphorylation and decrease its binding to microtubules resulting in increased levels of free tau, which is thought to promote its aggregation (19).

With tau as a key molecular player in AD and tauopathies, understanding the physiological role of tau is crucial for understanding its role in pathological conditions and the downstream effects of the loss- or gain-of tau function. Over the past decade, multiple studies have focused on physiological and pathological roles for tau beyond those related to microtubule stabilization (20). Tau is enriched in neuronal axons, with lower levels detected in the plasma membrane, dendrites, and dendritic spines, with a differential spatial distribution of tau isoforms (21). However, tau mislocalization in dendritic spines is known to cause synaptic dysfunction independently of neurodegeneration (22) and somatodendritic accumulation of tau occurs in AD (23). Studies from *Tau* KO mice have shown that loss of tau does not lead to gross behavioral or neuronal changes in young mice. However, tau does modulate synaptic plasticity. *Tau* KO mice have deficits in long-term potentiation and long-term depression (LTD) (24, 25). Characterization of the tau interactome in the mouse brain identified proteins involved in synaptic vesicle cycling and postsynaptic receptor trafficking (26). Yet, a mechanism for the physiological role of tau in regulating synaptic plasticity has not been clearly elucidated. Prior research also demonstrates that tau regulates N-methyl-D-aspartate (NMDA) receptor function by targeting Fyn tyrosine kinase to the post-synaptic density, where it phosphorylates NMDA receptors (27). Tau also contributes to the stability of α-amino-3-hydroxy-5-methyl-4-isoxazolepropionic acid (AMPA) receptors through its interaction with the ATPase NSF (26).

The activity-regulated cytoskeleton-associated protein (Arc) is an immediate early gene that regulates diverse forms of synaptic plasticity, memory, and learning (28, 29, 30, 31, 32). One mechanism through which Arc regulates synaptic plasticity is by promoting the endocytosis of AMPA receptors through interactions with members of the endocytic machinery; endophilin-2/3, dynamin 2, and AP-2 that dominantly depends on its N-terminal region referred to as the endophilin-binding (EB) domain (31, 33, 34).

Arc is upregulated during learning in the hippocampus (35) and is rapidly turned over, mainly through its ubiquitination and degradation by the ubiquitin-proteasome system (36, 37, 38, 39, 40). This type of posttranslational destabilization has been identified as a key mechanism for regulating group 1 metabotropic glutamate receptor-mediated LTD and spatial reversal learning (41). Arc is also removed by the autophagy-lysosomal pathway (42) and can be degraded by noncanonical neuronal membrane-associated proteasomes (43, 44). Additionally, Arc undergoes several posttranslational modifications including, but not limited to, phosphorylation (45), sumoylation (46, 47, 48), palmitoylation (49), and acetylation (50).

Several studies have examined the role of Arc in AD pathology, mainly by examining the relationship between Arc and β-amyloid (51, 52, 53, 54, 55). However, there is a gap in understanding the relationship between Arc and tau. Given the role of Arc as a key regulator of synaptic plasticity and the recent implications of tau in regulating synaptic plasticity (56, 57, 58), we set out to determine if Arc might be affected by tau pathology. Here, we show that endogenous tau has a physiological role in regulating Arc, where Arc levels are increased in the hippocampus of *Tau* KO mice and dendrites of *Tau* KO primary hippocampal neurons. Conversely, overexpression of WT-tau but not P301L-tau led to Arc instability. Tau-induced Arc reduction was found to be proteasome-dependent. Unexpectedly, tau-dependent Arc degradation was not associated with increased Arc ubiquitination, lysosomal degradation, or other known Arc posttranslational modifications that included phosphorylation, acetylation, or sumoylation. However, tau-dependent degradation did require the EB domain of Arc. Tau-induced alterations of Arc were selective to primary hippocampal dendrites and associated with increased surface GluA1-containing AMPA receptors in dendrites and the soma. Our findings highlight a unique role of WT-tau in spatially and noncanonically regulating Arc removal, with hints of Arc endocytic targeting involved in this process.

## Results

**Figure 1:**
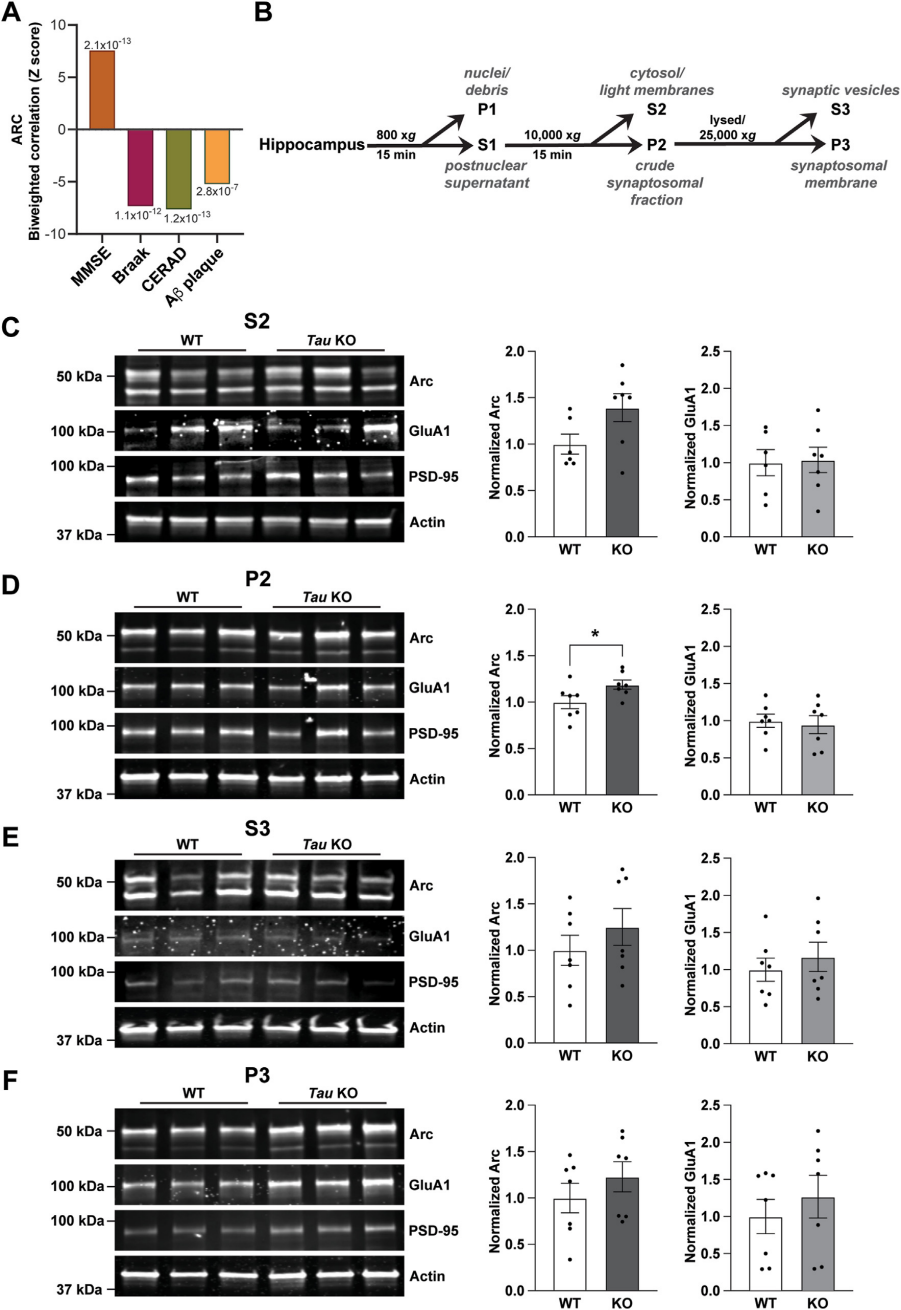
**Arc is increased in the hippocampus of *Tau* KO mice.***A*, Arc protein levels positively correlate with cognitive performance score and negatively correlate with AD progression scores. The mini-mental state examination (MMSE) indicates cognitive performance score, whereas the Consortium to Establish a Registry for Alzheimer’s Disease (CERAD) and Braak scores indicate AD progression. *B*, biochemical fractionation scheme. *C*, representative western blots showing Arc, GluA1, PSD-95, and Actin in the cytosol and light membrane fraction (S2). *D*, representative western blots showing Arc, GluA1, PSD-95, and Actin in the crude synaptosomal fraction (P2). Arc was significantly higher in the P2 fraction. Unpaired *t* test, t = 2.217, df = 12, *p* = 0.0467. *E*, representative western blots showing Arc, GluA1, PSD-95, and Actin in the synaptic vesicle fraction (S3). *F*, representative western blots showing Arc, GluA1, PSD-95, and Actin in the lysed synaptosomal membrane fraction (P3). No differences were found in GluA1 levels within any of the fractions. N = 6 animals per genotype, balanced for sex. AD, Alzheimer's disease; Arc, activity-regulated cytoskeleton-associated protein; PSD, postsynaptic density protein. ∗*p* < 0.05.

Numerous studies have demonstrated dysregulation of Arc in AD. For example, Arc levels are found to be elevated in the medial frontal cortex of AD patients and in the hippocampus of β-amyloid mouse models (55, 59). Upon re-analysis of the brain proteome, we found that protein levels of Arc correlated with cognitive performance in humans. Reductions in Arc were strongly correlated with AD status and Braak stages along with Amyloid levels (Fig. 1*A*) (60). Given previous studies on Arc regulation with β-amyloid, we sought to investigate a potential relationship between Arc and tau, which is highly upregulated and is another hallmark of AD pathology. To understand the endogenous regulation of tau on Arc, we measured Arc protein in *Tau* KO mice, which lack the *Mapt* gene that encodes for Tau. Knock-out of *Mapt* was confirmed by genotyping and the absence of Tau protein (Fig. S1, *A* and *B*). We next compared Arc in hippocampi harvested from 3-month-old *Tau* KO mice and their WT littermates. Surprisingly, Arc was significantly higher in total hippocampal lysates of *Tau* KO mice than WT (Fig. S1*C*; Arc, unpaired *t* test t = 2.42, df = 12, *p* = 0.032). Given the differential localization of Tau and Arc, we asked if this increase was specific to a neuronal compartment. We biochemically fractionated protein homogenates from the hippocampus using serial centrifugations (Fig. 1*B*). To demonstrate the effectiveness of our fractionation method, we analyzed GluA1 and the postsynaptic density protein-95 (PSD-95) in isolated fractions. As expected, there was an increase in GluA1 and PSD-95 in the crude synaptosomal fraction (P2) and the lysed synaptosomal membrane fraction (P3) compared to the cytosolic fraction (S2) (Fig. S1*D*). Arc was significantly elevated in the P2 but not the S2 fraction, although there was a strong trend towards upregulation of Arc in the S2 fraction (Fig. 1*C*, Arc in S2 t = 2.061, df = 11, *p* = 0.0638; Fig. 1*D*; Arc in P2, unpaired *t* test t = 2.217, df = 12, *p* = 0.0467). No significant differences in Arc were found in the P3 and the synaptic vesicle (S3) fractions (Fig. 1*E*; Arc in S3, unpaired *t* test t = 0.9787, df = 12, *p* = 0.347; Fig. 1*F*; Arc in P3, unpaired *t* test t = 1.005, df = 12, *p* = 0.3349). Differences in GluA1 were not observed in any of the fractions from *Tau* KO mice.

**Figure 2:**
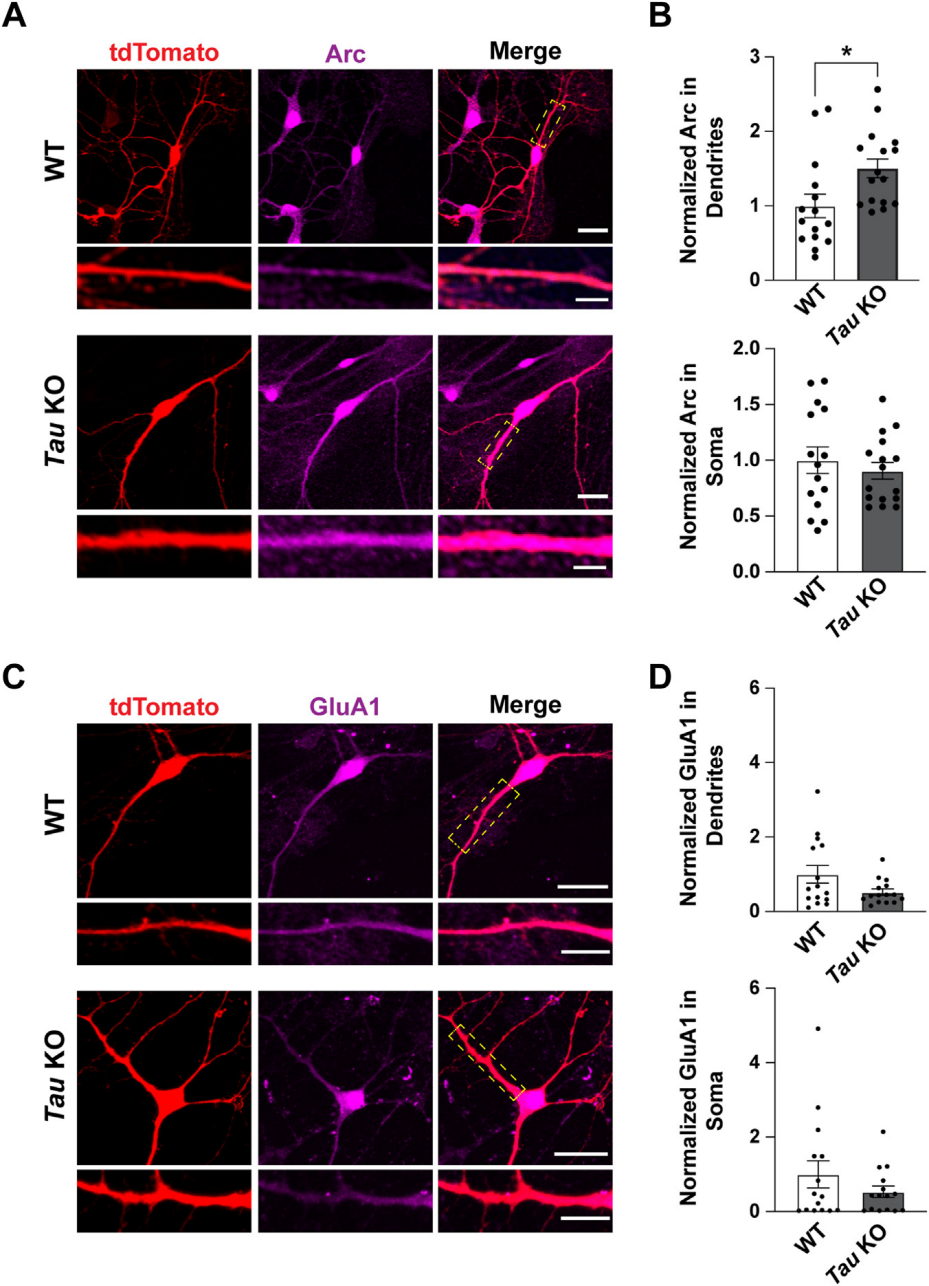
**Activity-dependent increase of Arc in dendrites in *Tau* KO neurons is not associated with significant changes in surface GluA1 levels.***A*, representative images of primary hippocampal neurons from WT and *Tau* KO littermates transfected at DIV 9 with tdTomato to outline neuron morphology, treated with 2 μM TTX for 4 h, and fixed at DIV 10. Scale bar represents 20 μm. Scale bar in selected dendrites represents 5 μm. *B*, quantification of Arc showing significantly higher levels in dendrites but not the soma. Unpaired *t* test for Arc in dendrites, t = 2.517, df = 29, *p* = 0.0176; unpaired *t* test for Arc in soma, t = 0.677, df = 29, *p* = 0.504. n = 15 to 16 neurons from two independent biological replicates. *C*, representative images of primary hippocampal neurons from WT and *Tau* KO littermates transfected at DIV 12 with tdTomato to outline neuron morphology, treated with 2 μM TTX for 4 h, and fixed at DIV 13. Scale bar represents 20 μm. Scale bar in selected dendrites represents 10 μm. *D*, quantification of GluA1 showing no significant differences between WT and *Tau* KO in soma nor dendrites. Mann Whitney test for GluA1 in dendrites, *p* = 0.32; Mann Whitney test for GluA1 in soma *p* = 0.65, n = 15 neurons from two independent biological replicates. Arc, activity-regulated cytoskeleton-associated protein; TTX, tetrodotoxin. ∗*p* < 0.05.

Since the increase in Arc was specific to the crude synaptosomal fraction, we investigated the spatial regulation of Arc by tau in primary hippocampal neuron cultures from WT and *Tau* KO littermates where GFP was used as a cell fill to outline neuronal morphology. While basal levels of Arc were unchanged between WT and *Tau* KO neurons, Arc remained elevated in *Tau* KO neurons selectively in dendrites upon manipulation of synaptic activity following blockade of action potentials with tetrodotoxin (TTX) (Fig. S2). Consistent with our hippocampal subcellular fractionation findings, Arc was selectively upregulated in dendrites and not the soma (Fig. 2, *A* and *B*; unpaired *t* test for Arc in dendrites, t = 2.517, df = 29, *p* = 0.0176; unpaired *t* test for Arc in soma, t = 0.677, df = 29, *p* = 0.504). These findings suggest that tau plays a physiological role in the activity-dependent regulation of Arc selectively in dendrites. Given the relationships between Arc and regulation of AMPA receptor synaptic scaling (31), we also quantified surface GluA1 in WT and *Tau* KO primary hippocampal neurons. While there was an average reduction of GluA1 in *Tau* KO neurons, there were no significant differences in GluA1 levels in soma or dendrites (Fig. 2, *C* and *D*, Mann Whitney test for GluA1 in dendrites, *p* = 0.32; Mann Whitney test for GluA1 in soma *p* = 0.65).

**Figure 3:**
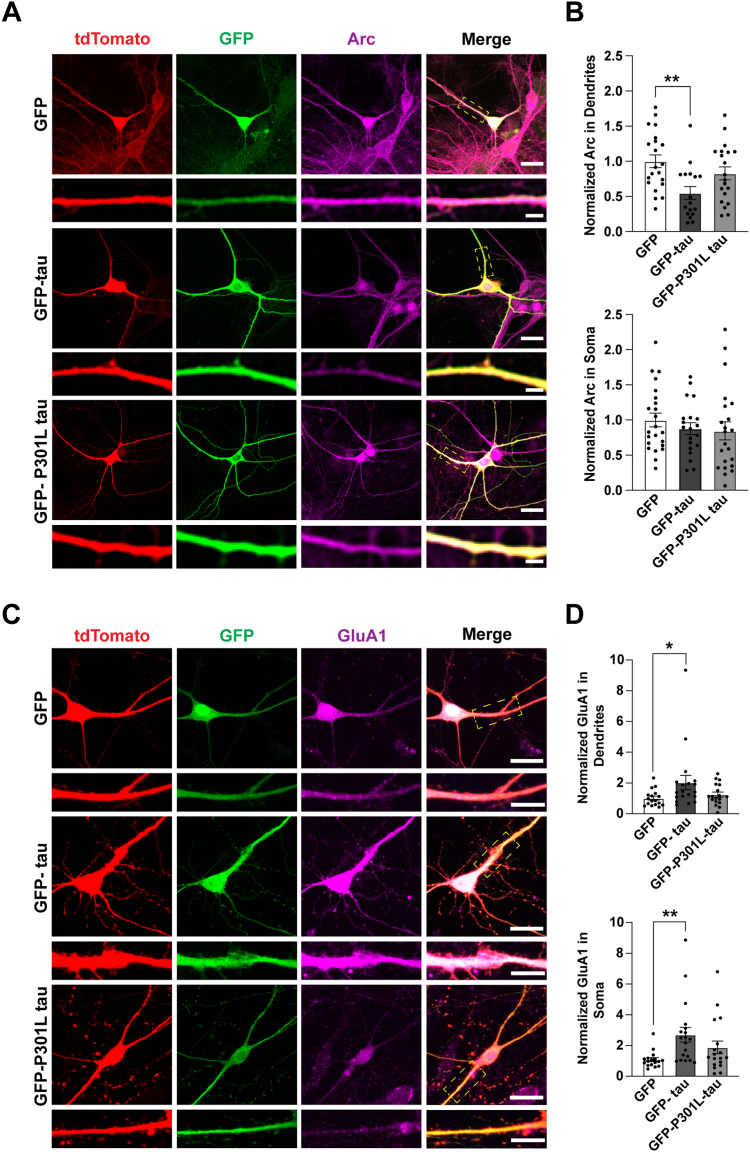
**GFP-tau but not GFP-P301L tau selectively reduces Arc in dendrites and correspondingly increases surface GluA1 in primary hippocampal neurons.***A*, primary hippocampal neurons transfected with GFP, GFP-tau, or GFP-P301L tau and treated with 2 μM TTX for 4 h. Tdtomato was used to outline neuron morphology. Scale bar represents 20 μm. Scale bar in selected dendrites represents 5 μm. *B*, quantification of Arc protein (*magenta*) in soma and dendrites showing a decrease in Arc selectively in dendrites with GFP-tau but not with GFP-P301L-tau. One-way ANOVA in dendrites F(2,55) = 5.885, *p* = 0.0048, Tukey’s post-hoc GFP *versus* GFP-Tau *p* = 0.0023; one-way ANOVA in soma F (2, 62) = 0.62, *p* = 0.54. n = 16 to 21 neurons from three independent biological replicates. *C*, primary hippocampal neurons overexpressing GFP, GFP-tau, and GFP-P301L tau and treated with 2 μM TTX for 4 h. TdTomato was used to outline neuron morphology. Neurons were fixed and then immunostained with an anti-GluA1 antibody. Scale bar represents 20 μm. Scale bar for selected dendrites represents 10 μm. *D*, quantification of surface GluA1 (*magenta*) in the soma and apical dendrites. Kruskal–Wallis test for dendrites, *p* = 0.0458; Kruskal–Wallis test for soma, *p* = 0.0034. n = 17 to 19 neurons from three independent biological replicates. Arc, activity-regulated cytoskeleton-associated protein; GFP-tau, GFP-tagged tau; TTX, tetrodotoxin. ∗*p* < 0.05, ∗∗*p* < 0.005.

We next asked if high levels of tau, similar to those observed in tauopathies would also affect Arc in neurons. GFP-tagged tau (GFP-tau) was overexpressed in WT primary hippocampal neurons and then treated with TTX. Neurons were fixed and endogenous Arc levels were quantified (Fig. 3*A*). In contrast to the increase of Arc in the dendrites of *Tau* KO mice, Arc was selectively decreased in dendrites upon GFP-tau overexpression. We also investigated the effect of GFP-P301L tau overexpression on Arc. P301L-tau is a missense single-point mutation located on the R2 MTBD that substitutes proline for leucine and has been commonly used to model AD pathology (17). However, unlike GFP-tau, overexpression of GFP-P301L tau did not affect Arc in soma or dendrites (Fig. 3*B*; One-way ANOVA in dendrites F(2,55) = 5.885, *p* = 0.0048, Tukey’s post hoc GFP *versus* GFP-Tau *p* = 0.0023; one-way ANOVA in soma F (2, 62) = 0.62, *p* = 0.54).

Given the role of Arc in regulating AMPA receptor trafficking (33), we hypothesized that the tau-mediated decrease of Arc in dendrites may result in alterations in activity-dependent AMPA receptor endocytosis and consequently lead to an increase in surface AMPA receptor levels. To test this, we overexpressed GFP-tau and GFP-P301L tau in primary hippocampal neurons and quantified surface GluA1-containing AMPA receptors (Fig. 3*C*). GluA1 staining in neurons overexpressing GFP-tau had a smoother appearance, unlike the punctate distribution in neurons overexpressing GFP and GFP-P301L tau. As expected, GFP-tau overexpression led to an increase in surface GluA1; however, this effect was not selective to dendrites, as overexpression also led to an increase of surface GluA1 in the soma (Fig. 3*D*, Kruskal–Wallis test for dendrites, *p* = 0.0458, Dunn’s multiple comparisons test, GFP *versus* GFP-tau *p* = 0.0415; Kruskal–Wallis test for soma, *p* = 0.0034, Dunn’s multiple comparisons test, GFP *versus* GFP-tau *p* = 0.0023). To determine if changes in GluA1 were associated with alterations in excitatory synapses, we quantified dendritic spine densities in neurons expressing GFP, GFP-tau, and GFP-P301L tau treated with TTX. However, we found no significant differences in spine density compared to GFP alone (Fig. S2, Ordinary one-way ANOVA F (2, 50) = 0.1543, *p* = 0.8574). These findings suggest that WT-tau but not P301L tau overexpression decreases Arc and that overexpression of WT-tau subsequently increases surface expression of GluA1-containing AMPA receptors.

**Figure 4:**
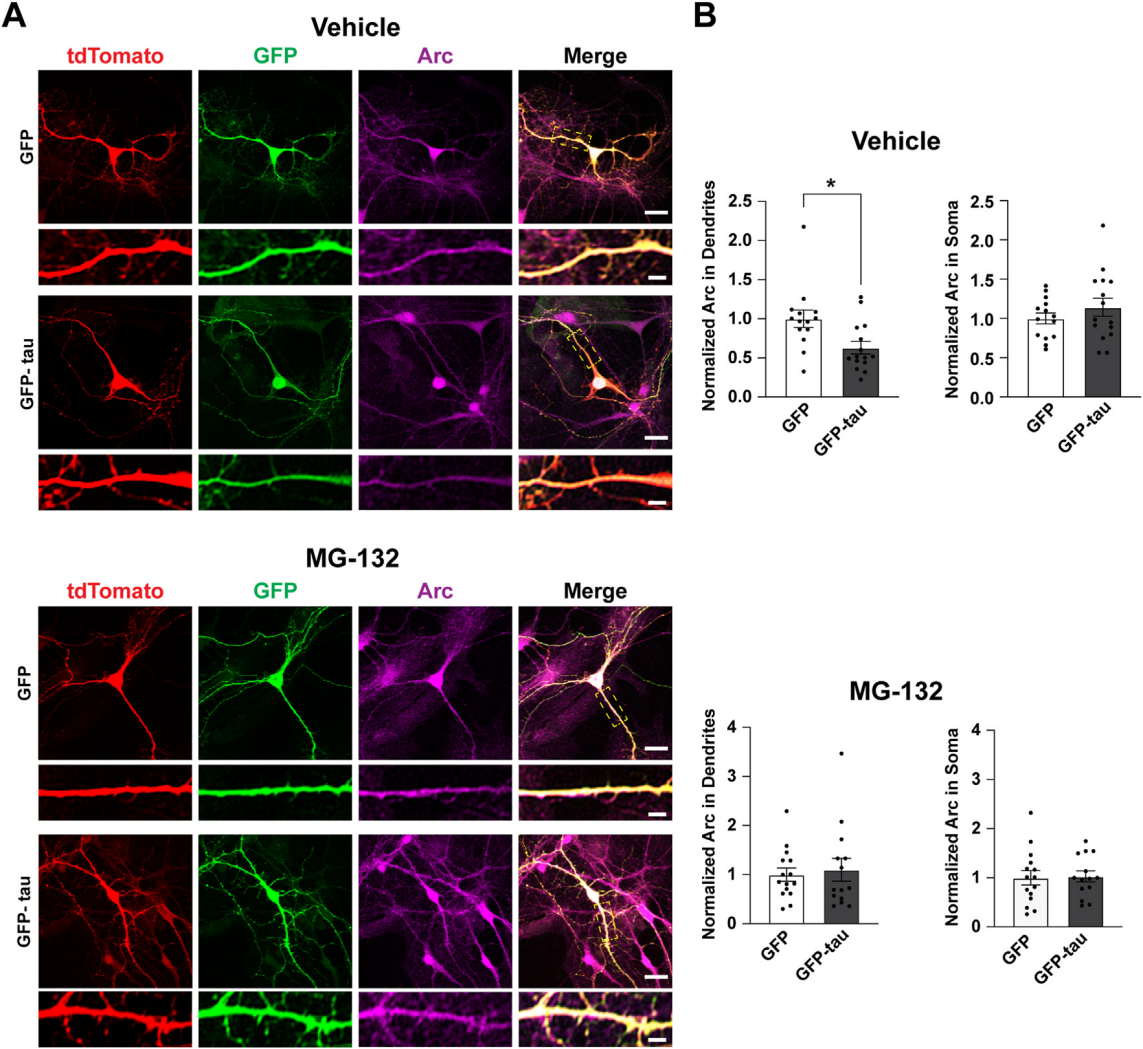
**Tau-induced decreases in dendritic Arc are proteasome-sensitive.***A*, primary hippocampal neurons overexpressing GFP or GFP-tau. tdTomato was used to outline neuron morphology. Cells were treated with TTX and then treated with either Vehicle (DMSO) or MG-132 (10 μM) for 4 h. Scale bar represents 20 μm. Scale bar in selected dendrites represents 5 μm. *B*, quantification of Arc protein (*magenta*) in the soma and apical dendrites showing a decrease in Arc selectively in dendrites in cultures treated with Vehicle but not in cultures treated with MG-132. Unpaired *t* test DMSO in dendrites t = 2.72, df = 27, *p* = 0.011; unpaired *t* test MG-132 in dendrites t = 0.3814, df = 27, *p* = 0.706; unpaired *t* test DMSO in soma t = 1.044, df = 27, *p* = 0.306; unpaired *t* test MG-132 in soma t = 0.16, df = 27, *p* = 0.874. n = 14 to 15 neurons from two independent biological replicates. Arc, activity-regulated cytoskeleton-associated protein; GFP-tau, GFP-tagged tau; TTX, tetrodotoxin. ∗*p* < 0.05.

We sought to determine the mechanism through which tau regulates activity-induced Arc expression. One possibility is that tau could be regulating Arc stability at the posttranslational level. Ubiquitination of Arc is a modification that facilitates its degradation by the proteasome, which is a major pathway for its posttranslational removal (36, 37, 40). Therefore, we asked whether the reduction of Arc by tau was dependent on proteasome activity. To determine if tau-dependent modulation of Arc was proteasome-sensitive, primary hippocampal neurons overexpressing GFP-tau were treated with TTX followed by the proteasome inhibitor MG-132 and Arc was quantified (Fig. 4*A*). In Vehicle-treated neurons, Arc was significantly decreased upon GFP-tau overexpression but not in MG-132–treated neurons (Fig. 4*B*; Unpaired *t* test DMSO Control in dendrites t = 2.72, df = 27, *p* = 0.011; unpaired *t* test MG-132 in dendrites t = 0.3814, df = 27 *p* = 0.706; unpaired *t* test DMSO in soma t = 1.044, df = 27, *p* = 0.306; unpaired *t* test MG-132 in soma t = 0.16, df = 27, *p* = 0.874). Our findings support the notion that activity-dependent tau modulation of Arc is selective for dendrites and is proteasome-sensitive.

**Figure 5:**
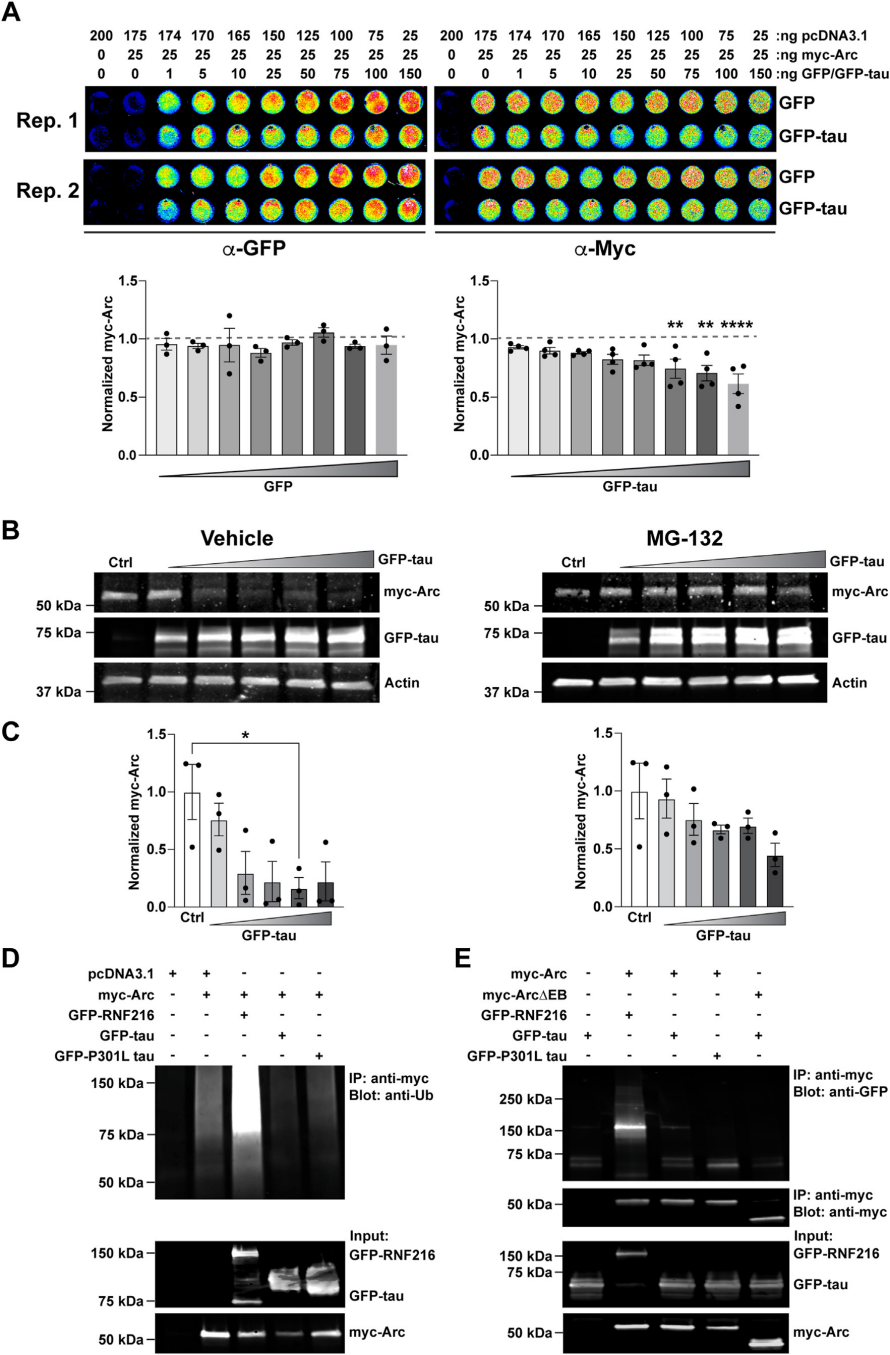
**Tau modulation of Arc in HEK293 cells is proteasome-sensitive but does not increase Arc ubiquitination.***A*, *top*, Titration of GFP-tau but not GFP reduces myc-Arc expression in HEK293 cells. HEK 293 cells were transfected with myc-Arc and increasing amounts of GFP or increasing amounts of GFP-tau. pcDNA3.1 was used as a DNA filler to keep the amount of transfected DNA between titration conditions identical. Cells were fixed, permeabilized, and then stained using anti-GFP (*left*) and anti-myc (*right*) antibodies. *Bottom*, quantification of GFP and myc-Arc in cells with increasing concentrations of GFP or GFP-tau. One-way ANOVA for GFP F (8, 18) = 0.6185, *p* = 0.7516; GFP-tau F (8, 27) = 5.560, *p* = 0.0003. Dunnett’s multiple comparisons test ∗∗*p* < 0.005, ∗∗∗∗*p* < 0.0001. n = 3 to 4. *B*, following transfection, HEK293 cells were treated with either Vehicle (DMSO) or MG-132 (10 μM) for 4 h. *Left*, *r*epresentative western blots showing myc-Arc with increasing concentrations of GFP-tau in DMSO-treated HEK293 cells (0, 0.25, 0.5, 0.75, 1, and 1.5 μg). Actin was used as a loading control. *Right*, representative western blots showing myc-Arc with increasing concentrations of GFP-tau in MG-132–treated HEK293 cells (0, 0.25, 0.5, 0.75, 1, and 1.5 μg). Actin was used as a loading control. pcDNA3.1 was used as a DNA filler to keep the amount of transfected DNA between titration conditions identical. *C*, quantification of myc-Arc normalized to Actin showing a significant decrease in Arc with increasing tau in vehicle-treated cells (*left*) but not in the MG-132–treated cells (*right*). One-way ANOVA for DMSO control, F (5, 12) = 4.03, *p* = 0.022; one-way ANOVA for MG-132, F (5, 12) = 1.9, *p* = 0.15. n = 3. *D*, *top*, ubiquitin assay showing no enhancement in Arc ubiquitination when co-expressed with tau or P301L tau. RNF216 was used as a positive control. *Bottom*, input showing expression of myc-Arc, GFP-RNF216, GFP-tau, and GFP-P301L tau. *E*, coimmunoprecipitation assay showing pulldown of myc-Arc or myc-Arc ΔEB with an anti-myc antibody and immunoblotting with an anti-GFP antibody. GFP-RNF216 was used as a positive control. Arc, activity-regulated cytoskeleton-associated protein; EB, endophilin-binding; GFP-tau, GFP-tagged tau. ∗*p* < 0.05.

We next turned to HEK293 cells, which are more amenable to performing biochemical studies to further elucidate the possible multitude of mechanisms through which WT-tau regulates Arc. First, we determined if we could create conditions that allowed us to observe changes in Arc levels, like our findings in neurons. To do this, we used a 96-well plate in-cell western format (61, 62) to directly compare a series of GFP control or GFP-tau titrations with myc-Arc in HEK293 cells. GFP or GFP-tau were titrated at increasing concentrations, and filler DNA (pcDNA3.1) was used to keep the total amount of DNA transfected in the cells constant. Like observations with endogenous Arc in primary hippocampal neurons, myc-Arc was reduced upon increasing concentrations of GFP-tau whereas titration of the GFP control had no significant effect (Fig. 5*A*). To determine if reductions in Arc levels were proteasome-sensitive, cells overexpressing myc-tagged Arc and increasing concentrations of GFP-tau were treated with the proteasome inhibitor MG-132. Similar to observations above, myc-Arc was reduced upon increasing concentrations of GFP-tau but this effect was not observed in cells treated with MG-132 (Fig. 5*C*; one-way ANOVA for DMSO, F (5, 12) = 4.03, *p* = 0.022, Tukey’s post-hoc test 0 *versus* 1 μg: *p* = 0.046; one-way ANOVA for MG-132, F (5, 12) = 1.9, *p* = 0.15). We also overexpressed myc-tagged Arc with increasing concentrations of GFP-P301L tau and found that this mutation did not reduce myc-Arc with increasing GFP-P301L tau, which was also similar to our experiments in primary hippocampal neurons. (Fig. S4*A*, One-way ANOVA, F (5, 18) = 0.3018, *p* = 0.3).

Studies have shown that Arc can be ubiquitinated by the E3 ligases RNF216, UBE3A, and CHIP, which induces its rapid degradation by the proteasome (36, 37, 39). Since protein ubiquitination dominantly occurs on lysine residues, we tested reported Arc ubiquitination sites for RNF216 and UBE3A on lysines which were mutated to arginine to prevent ubiquitination (K55R/K136R/K268R/K269R/K293R, myc-Arc5KR) (36, 37) (Fig. S4*B*). Surprisingly, cotransfection of myc-Arc5KR with increasing amounts of GFP-tau still led to a reduction in Arc that was similar in magnitude to myc-Arc WT (Fig. S4*C*; one-way ANOVA F = (5, 18) = 6.4, *p* = 0.0014, Tukey’s posthoc 0 *versus* 0.5 μg = 0.02, 0 *versus* 0.75 μg *p* = 0.0175, 0 *versus* 1 μg *p* = 0.0015, 0 *versus* 1.5 μg *p* = 0.0016). These findings suggested that preventing Arc ubiquitination at these sites does not interfere with tau-mediated Arc reduction. To determine if the tau-mediated decrease in Arc involved ubiquitination, we measured myc-Arc ubiquitination in the presence of GFP-tau or GFP-P301L tau after treatment with MG-132 to trap Arc-ubiquitinated products. While RNF216 robustly increased myc-Arc ubiquitination, this was not observed upon GFP-tau or GFP-P301L tau overexpression (Fig. 5*D*). To test if Arc could interact with WT-tau, we performed co-immunoprecipitation assays with WT or P301L-tau. While RNF216 efficiently co-immunoprecipitated with Arc, interaction with WT or P301L-tau was not different compared to background binding control. Moreover, the interaction between tau and Arc did not change upon deletion of the EB domain of Arc (myc-Arc ΔEB), which is a domain that targets Arc to endosomes (33) (Fig. 5*E*). Taken together, these experiments suggest that even though tau modulation of Arc is proteasome-sensitive, it does not appear to be through an interaction with Arc or through enhancing Arc ubiquitination by RNF216 or UBE3A, which indicates that tau might be utilizing proteasome-sensitive noncanonical methods for Arc removal.

One alternative possibility to our proteasome inhibition studies could be related to reports of MG-132–blocking late phases of the lysosomal pathway, another method cells use to degrade proteins (63). Given that ubiquitinated Arc is also removed by the autophagy-lysosomal system in neurons (42), we asked if tau decreases Arc by lysosome-dependent degradation. We treated cells overexpressing myc-Arc and GFP-tau with the lysosome inhibitors Leupeptin and ammonium chloride for 6 h before harvest (Fig. S5*A*). We confirmed inhibition of lysosomal activity by blotting for markers of autophagic flux—MAP1LC3B (hereafter LC3) and p62/SQSTM1 (64). There was a significant increase in the autophagosome-bound form of LC3 – LC3-II (Fig. S5*B*; unpaired *t* test, t = 12.52, df = 22, *p* < 0.0001) and the autophagosome substrate p62/SQSTM1 (unpaired *t* test, t = 10.54, df = 22, *p* < 0.0001) in cells treated with lysosome inhibitors as compared to vehicle-treated cells. Surprisingly, we did not observe the expected increase in Arc protein (Fig. S5*C*; Unpaired *t* test, t = 0.7087, df = 10, *p* = 0.4947). Nevertheless, Arc was decreased upon GFP-tau overexpression in both vehicle and inhibitor-treated conditions (Fig. S5*C*; unpaired *t* test for vehicle control, t = 10.6, df = 10, *p* < 0.0001; unpaired *t* test for inhibitors, t = 5.585, df = 10, *p* = 0.0002). These findings demonstrate that tau modulation of Arc is not lysosome-dependent.

Given the ability of overexpressed tau to form insoluble aggregates (65, 66), we next asked if tau may be precipitating Arc into insoluble aggregates that could not be extracted in the radioimmunoprecipitation assay (RIPA)-soluble fraction. We extracted proteins in the RIPA-insoluble fraction using formic acid, which has been successfully used to extract tau aggregates (67, 68). While GFP-tau was detected in the insoluble fraction, Arc was not (Fig. S5*D*), indicating that tau-dependent decreases in Arc are not due to its precipitation into insoluble aggregates.

**Figure 6:**
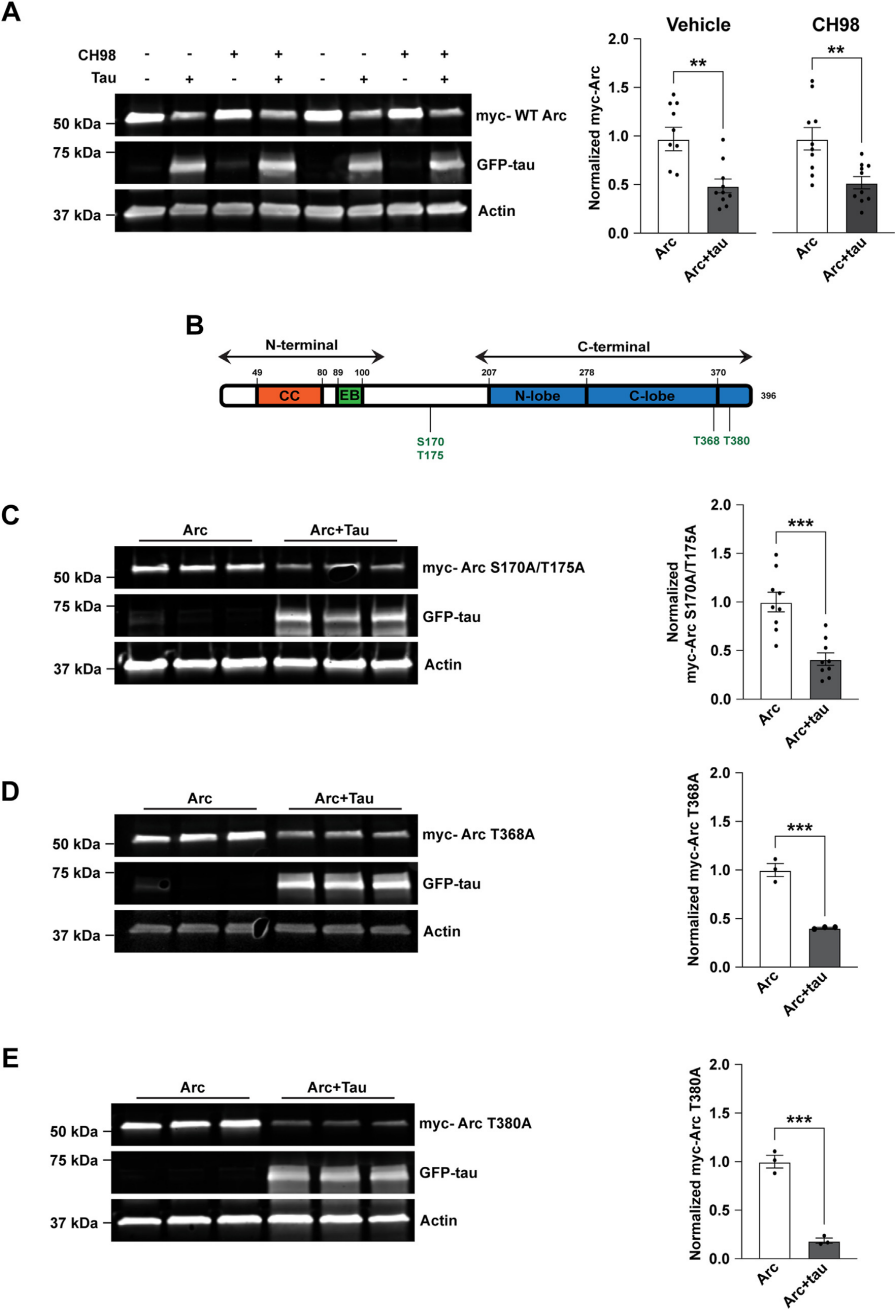
**Tau-induced Arc removal does not depend on GSK3α/β-dependent Arc phosphorylation.***A*, *left*, representative western blots showing myc-Arc expressed alone or with GFP-tau. Cells were treated with Vehicle (Water) or CH98 (1–2 μM) for 4 h. Actin was used as a loading control. *Right*, quantification of myc-Arc showing a significant decrease with co-expression of GFP-tau after treatment with Vehicle (unpaired *t* test, t = 3.450, df = 18, *p* = 0.0029) or CH98 (unpaired *t* test, t = 3.417, df = 18, *p* = 0.0031). n = 9. *B*, schematic showing the structure of Arc with the location of mapped GSK3α/β phosphorylation sites S170, T175, T368, and T380. *C*–*E*, *left*, representative western blots showing myc-Arc S170A/T175A, myc-Arc T368A, or myc-Arc T380A with Arc phosphorylation sites mutated to Alanine expressed alone or with GFP-tau. Actin was used as a loading control. *Right*, Quantification of myc-Arc S170A/T175A, myc-Arc T368A, or myc-Arc T380A showing a significant decrease when co-expressed with tau. Unpaired *t* test for Arc S170A/T175A, t = 4.913, df = 16, *p* = 0.0002; unpaired *t* test for Arc T368A, t = 8.714, df = 4, *p* = 0.001; unpaired *t* test for Arc T380A, t = 11.59, df = 4, *p* = 0.0003. n = 9 for S170A/T175A, n = 3 for T368A and T380A. Arc, activity-regulated cytoskeleton-associated protein; GFP-tau, GFP-tagged tau. ∗∗*p* < 0.05, ∗∗∗*p* < 0.001.

One recent study suggested that Arc phosphorylation by GSK3α/β can enhance its removal by the proteasome (45). We treated cells co-expressing myc-Arc and GFP-tau with the GSK3α/β inhibitor CHIR 98014 (CH98) for 4 h before harvest (Fig. 6*A*). myc-Arc decreased with GFP-tau overexpression in both vehicle and the CH98-treated condition (Fig. 6*A*; unpaired *t* test for vehicle control, t = 3.450, df = 18, *p* = 0.0029; unpaired *t* test for CH98, t = 3.417, df = 18, *p* = 0.0031). It was also reported that Arc is phosphorylated on S170, T175, T368, and T380 by GSK3α/β (45) (Fig. 6*B*). We mutated these phosphorylation sites to generate myc-Arc S170A/T175A, myc-Arc T368A, and myc-Arc T380A. However, upon overexpression with GFP-tau (Fig. 6, *C*–*E*), all three of these myc-Arc phosphorylation mutants were still decreased (Fig. 6, *C*–*E*; Unpaired *t* test for Arc S170A/T175A, t = 4.913, df = 16, *p* = 0.0002; unpaired *t* test for Arc T368A, t = 8.714, df = 4, *p* = 0.001; unpaired *t* test for Arc T380A, t = 11.59, df = 4, *p* = 0.0003). Together, these experiments demonstrate that tau modulation of Arc is not mediated through GSK3α/β activity or GSK3α/β-dependent Arc phosphorylation.

**Figure 7:**
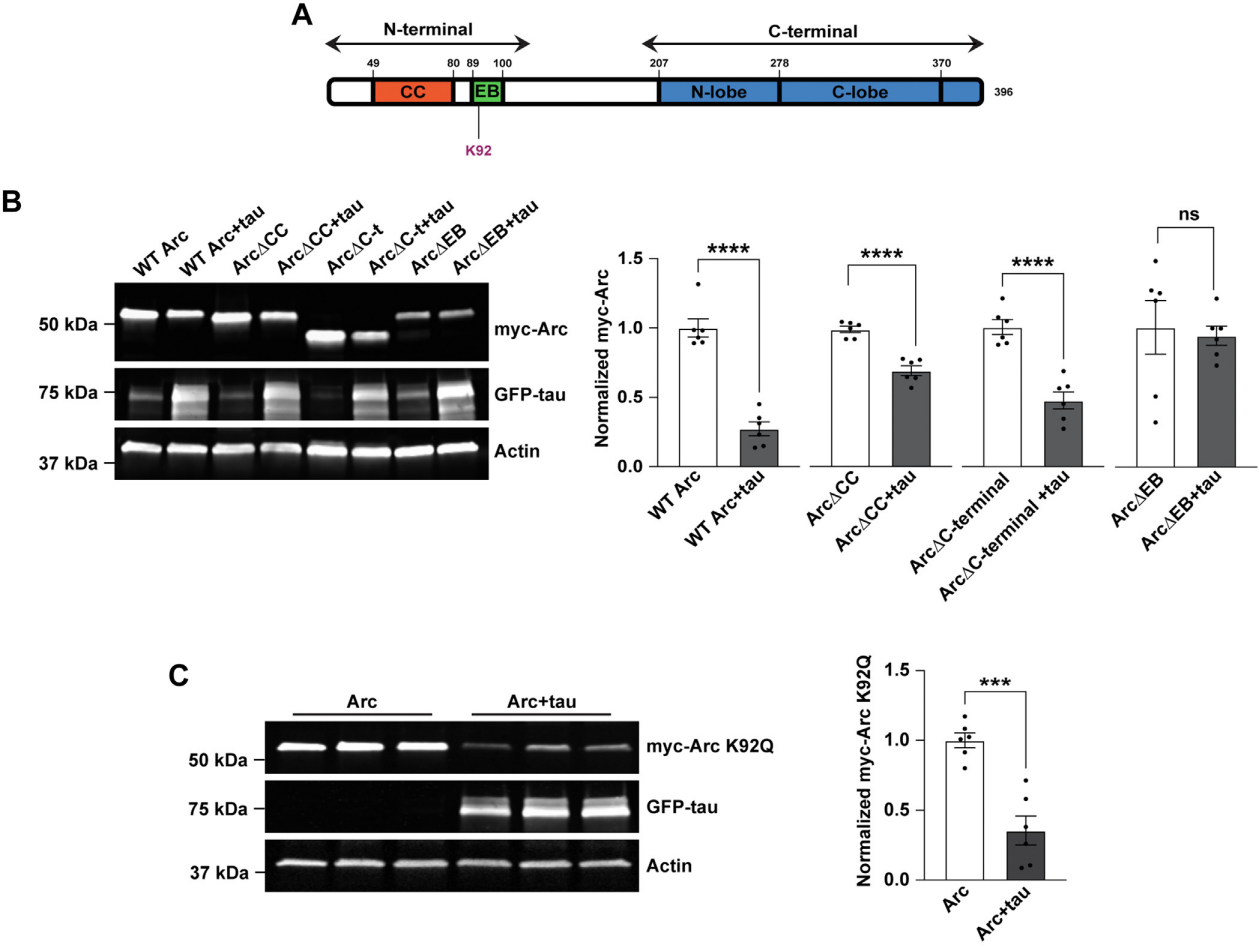
**The endophilin-binding domain of Arc is essential for its reduction by tau.***A*, schematic showing the structure of Arc, highlighting the coiled-coil (CC) domain and the endophilin-binding (EB) domain on the N terminus and the N- and C-lobe on the C terminus. The location of the K92 acetylation site is shown. *B*, *left*, Representative Western blot showing WT myc-Arc, myc-Arc ΔC-terminal (lacking the C-terminal domain), myc-Arc ΔCC (lacking the coiled-coil motif on the N-terminal domain), and myc-Arc ΔEB (lacking the endophilin-binding domain on the N-terminus) expressed alone or with GFP-tau. Actin was used as a loading control. *Right*, Quantification of WT myc-Arc, myc-Arc ΔC-terminal, myc-Arc ΔCC, and myc-Arc ΔEB. Only Arc ΔEB does not show a decrease with tau overexpression (Unpaired *t* test for WT Arc t = 8.857, df = 10, ∗∗∗∗*p* < 0.0001; unpaired *t* test for Arc ΔC-terminal t = 6.471, df =1 0, ∗∗∗∗*p* < 0.0001; unpaired *t* test for Arc ΔCC t = 7.076, df = 10, ∗∗∗∗*p* < 0.0001; unpaired *t* test Arc ΔEB t = 0.2956, df = 10, *p* = 0.774). n = 6. *C*, *left*, Representative western blots showing myc-Arc K92Q with the acetylation site K92 mutated to glutamine expressed alone or with GFP-tau. Actin was used as a loading control. *Right*, Quantification of myc-Arc K92Q showing a significant decrease when co-expressed with tau. Unpaired *t* test t = 5.54, df = 10, *p* = 0.0002. n = 6. Arc, activity-regulated cytoskeleton-associated protein; GFP-tau, GFP-tagged tau. ∗∗∗*p* < 0.001, ∗∗∗∗*p* < 0.0001.

The lack of effects of ubiquitin and phosphorylation-modifying sites to mediate Arc removal by tau prompted us to evaluate larger regions of Arc that might be necessary for tau-dependent reductions. We used myc-Arc constructs that lack specific domains of Arc; myc-Arc ΔC-terminal (lacking the C-terminal domain), myc-Arc ΔCC (lacking the coiled-coil motif on the N-terminal domain), and myc-Arc ΔEB (lacking the EB domain on the N terminus) (37) (Fig. 7*A*). We found that all the tested myc-Arc constructs were significantly decreased with tau except for the myc-Arc ΔEB (Fig. 7*B*; unpaired *t* test for WT Arc t = 8.857, df = 10, *p* < 0.0001; unpaired *t* test for Arc ΔC-terminal t = 6.471, df = 10, *p* < 0.0001; unpaired *t* test for Arc ΔCC t = 7.076, df = 10, *p* < 0.0001; unpaired *t* test Arc ΔEB t = 0.2956, df =1 0, *p* = 0.774). Cumulatively, these findings suggest that the EB domain of Arc is essential for its reduction by tau.

The Arc EB domain is an 11 amino acid sequence that is important for targeting Arc to endosomes (33). Recently, Arc was found to be acetylated at K24, K33, K55, and K92 which increases its stability (50). Of these sites, K92 falls within the EB domain (69, 70, 71, 72, 73, 74, 75, 76, 77, 78, 79, 80) (Fig. 7*A*). We hypothesized that tau could be decreasing Arc stability by interfering with its acetylation at K92. To test this hypothesis, we created the acetyl-mimetic myc-Arc K92Q, which increases the stability of Arc (50). However, the myc-Arc K92Q mutant was still reduced with GFP-tau overexpression suggesting that tau does not modulate Arc by interfering with its acetylation at K92 (Fig. 7*C*; unpaired *t* test t = 5.54, df = 10, *p* = 0.0002). Cumulatively, these findings suggest that tau regulation of Arc does not occur through canonical degradation pathways and known posttranslational modifications but does require its EB domain.

## Discussion

In this study, we evaluated the relationships between tau and Arc stability. Based on previous work, we hypothesized that tau overexpression may increase Arc levels given previous studies using AD mouse models (51, 52, 53, 54, 81, 82, 83). However, we found that the opposite was true. First, lower Arc levels were predicted to track with AD severity and overexpression of tau led to an activity-dependent reduction of Arc in hippocampal neurons. Moreover, this regulation appeared to be physiological, as knocking out *Tau* in hippocampal neurons led to an increase in Arc in hippocampal lysates and the crude synaptosomal subcellular fraction of *Tau* KO mice. Arc regulation by Tau was also found to be compartment-specific, as Arc was found to be elevated in neuronal dendrites of *Tau* KO primary hippocampal neurons treated with TTX. Overexpression of tau led to the opposite effect; reducing Arc selectively in neuronal dendrites. This effect was not present upon expression of the tau P301L mutation, which is highly prone to aggregation and has reduced binding to microtubules (19). The tau-dependent decrease of Arc in dendrites was also proteasome-sensitive, indicating that these effects are posttranslationally driven. The decrease of Arc with tau overexpression was also associated with an increase in the surface expression of the AMPA receptor subunit GluA1 in both soma and dendrites. In attempting to decipher the mechanism through which tau regulates Arc, we tested the role of numerous Arc posttranslational modifications in HEK293 cells that could be responsible for regulating tau-dependent Arc turnover. Despite dependence on the proteasome, tau regulation of Arc was independent of Arc ubiquitination, phosphorylation, acetylation, and lysosomal degradation mechanisms, suggesting that tau regulation of Arc occurs through noncanonical pathways. However, the EB domain of Arc was necessary for its modulation by tau, suggesting a role for Arc engagement with the endocytic machinery for tau-induced instability. Since tau mislocalization to dendritic spines causes synaptic dysfunction (22) and somatodendritic accumulation of Tau occurs in AD (23), our finding provides a potential molecular basis for synaptic dysfunction mediated through accumulation of tau in dendrites.

Several studies have shown a role for Arc in AD, mainly through links to amyloid-beta (Aβ). For example, both increases and decreases in Arc in the hippocampus and cortex were reported in several amyloid precursor protein mouse models (51, 52, 53, 54, 81, 82, 83) and it has been suggested that these changes occur in an age-dependent manner (59, 84). Arc levels are also increased in the medial prefrontal cortex of patients with AD (55). On the other hand, a mechanistic relationship between Arc and tau has been relatively understudied. While experience-driven Arc responses were found to be disrupted in the vicinity of plaques in the amyloid precursor protein/PS1 model, where neurons in the vicinity of amyloid plaques were less likely to respond, no similar effect was observed in the vicinity of tau NFTs in the rTg4510 mouse overexpressing P301L tau (85, 86). A recent study showed that tau elevated Arc1 in a *drosophila* AD model overexpressing R406W tau, a mutant linked to Frontotemporal dementia, demonstrating a role for Arc1 in neurodegeneration (87). While the conflicting results make it difficult to define a clear role for Arc in AD pathology, this can be attributed to differences in species, the disease models used, the stage of disease development, and the tissue type. Differences in levels of excitability between networks and brain areas can also explain some of the contradictions as Arc levels increase rapidly in excited synapses and upon exposure to learning experiences followed by its removal to return to its basal levels (37, 59, 88, 89, 90).

We show that tau modulation of Arc is proteasome-sensitive. A major pathway for Arc removal is through the ubiquitin-proteasome system (36, 37, 38, 39, 40). However, creating mutations of characterized Arc ubiquitination sites targeted by the E3 ligases RNF216 and UBE3A (Arc5KR) did not block Arc degradation (36, 37). Although we did not test for a role of CHIP, another E3 ligase that regulates the ubiquitination of both Arc and tau (39, 91), the possibility remains unlikely as we could not detect an enhancement of Arc ubiquitination upon tau overexpression. Moreover, tau modulation of Arc was not dependent on Arc phosphorylation or lysosomal degradation. In light of these findings, three alternative mechanisms can still be hypothesized. First, recent studies have found that Arc can be assembled into viral-like capsids and released into the extracellular space (92, 93, 94). It is possible that tau overexpression may result in the extracellular release of Arc capsids. Second, Arc is a substrate of the neuronal membrane proteasome, which is proteasome inhibitor–sensitive and utilizes a ubiquitin-independent mechanism for the degradation of ribosome-associated nascent Arc (43, 44). However, to our knowledge, HEK293 cells do not express the neuronal membrane proteasome, yet we found that tau overexpression was still able to reduce Arc in a proteasome-dependent manner. A final possibility is that Arc may be degraded by 20S uncapped proteasomes, which are MG-132–sensitive and can also function to degrade proteins independently of ubiquitination (95). Both neuronal and nonneuronal cells express 20S uncapped proteasomes at differing abundances (96, 97).

We show that the EB domain of Arc is required for modulation by tau. Structurally, Arc consists of two juxtaposed domains, a positively charged N-terminal domain and a negatively charged C-terminal domain. The N-terminal domain has several peptide-binding sites, including the EB and two long helices possibly forming a coiled-coil (98, 99). Arc mediates endocytosis of AMPA receptors through its interaction with endophilin at the EB domain (33). Given our findings requiring the EB of Arc, we investigated the possibility that tau overexpression could be interfering with Arc stability by disrupting the acetylation of Arc at K92 located within this domain (50), but an acetyl mimetic Arc mutant at this site (K92Q) did not prevent the decrease in Arc. Moreover, Arc did not co-immunoprecipitate with tau even when blocking its degradation with the proteasome inhibitor MG-132, suggesting an indirect or transient interaction between these two proteins. Arc binds endophilin, dynamin, and AP-2 to mediate endocytosis of AMPA receptors (33, 34). Recent findings show that tau (2N4R) has an extensive network of interactions with proteins that regulate endocytosis, including endophilin, AP-2, and dynamin (26, 100). Interestingly, expression of human tau (0N4R) induces de novo assembly of microtubules which interferes with endocytosis through sequestration of dynamin (69). Although we are unable to define a detailed mechanism for the modulation of Arc by tau, our results suggest the involvement of the endocytotic machinery, engaged by both Arc (*via* its EB domain) and tau (*via* its R2 MTBD), in this process. However, it is important to be careful with the interpretation of data from a heterologous expression system such as HEK293 cells. While we show that the decrease in Arc by tau is proteasome-sensitive in both neurons and HEK293 cells, the upstream mechanisms involved in targeting endogenous Arc to the proteasome in primary hippocampal neurons could potentially be different from mechanisms of removal of artificially expressed myc-Arc in HEK293 cells. This becomes particularly important in studies such as ours that evaluate alterations in the levels of artificially expressed proteins. While we controlled for the efficiency of transfection by keeping the amount of DNA transfected into the cell identical in all conditions by adding the filler plasmid pcDNA3.1 and counterbalancing GFP expression by using a GFP control plasmid from the same plasmid backbone as GFP-tau, there may be a potential confound in some of our conclusions from experiments conducted solely in HEK293 cells.

Consistent with the observed decrease in Arc specifically in hippocampal dendrites upon tau overexpression, we found that WT tau overexpression with modulating neural activity with TTX increases surface levels of GluA1 in the soma of primary hippocampal neurons and dendrites. Since surface GluA1 was also increased in the soma despite no detected change in Arc in that region, we propose that tau overexpression might mistarget GluA1-containing AMPA receptors to the soma due to altered trafficking. The physiological interactions of dendritic tau with synaptic proteins that regulate postsynaptic receptor trafficking and synaptic plasticity have been described (24, 26, 56, 70, 71). Importantly, we did not observe a change in surface GluA1 in *Tau* KO mouse synaptosomes and cultured primary hippocampal neurons. A study that investigated surface GluA1 levels in hippocampal neurons of *Tau* KO mice under different conditions showed no change in basal surface GluA1 in neurites but a decrease in total surface GluA1 compared to WT controls (26). Tau interacts with several proteins that regulate AMPA receptor trafficking such as NSF and PICK1 (26, 71), and *Tau* knockout neurons show a rapid reduction in the number of GluA2 puncta after NMDA treatment (72). Additionally, tau plays a role in the postsynaptic targeting of the Src kinase Fyn, which regulates the activation of NMDA receptors (56), and consequently affects AMPA receptor trafficking (73). Thus, the observed results could be a net effect of the interaction of tau with several proteins. The sequestration of these proteins could lead to Arc instability. In contrast, we did not observe a change in surface GluA1 with P301L tau overexpression in the soma or dendrites of hippocampal neurons. Hoover *et al.* (22) reported higher levels of P301L tau in postsynaptic density proteins isolated from rTg4510 mice overexpressing P301L tau compared to those isolated from rTg21221 mice overexpressing WT tau (0N4R). rTgP301L neurons showed lower spine GluA1 levels than neurons from rTgWT mice at days *in vitro* (DIV) 21 to 35. In our study, we did not find any changes in dendritic spine densities between WT and P301L tau treated with TTX. These discrepancies may be due to acute *versus* chronic overexpression and differences in manipulating synaptic activity. Additionally, the insertion of the transgene *MAPT P301L* in the rTg4510 model disrupts the *fibroblast growth factor 14* (*Fgf14*) gene, which has been shown to contribute to the neurodegeneration observed in the rTgP301L mouse model, which may have inadvertently resulted in off-target effects on surface GluA1 and spine densities (74, 75).

While different tau isoforms and mutants are used interchangeably to model AD and other tauopathies, our study adds to the growing body of literature that emphasizes the differences in protein interactions between tau and its mutants, which would suggest that they have different roles in the cell as well as different contributions to disease pathogenesis (21, 76, 77, 78, 100). The six isoforms of tau are differentially expressed throughout development, with the ratio of 3R to 4R tau in the adult human brain roughly equal to one (8). Tau isoforms further have distinct biochemical properties such as different propensities for aggregation, with those containing 4R assembling 2.5 to 3 times faster than 3R isoforms (79). In AD, NFTs contain all six isoforms, while in other tauopathies, tangles may predominantly have 3R or 4R tau (80). On the other hand, the majority of *MAPT* mutations, including P301L, are associated with FTD. However, P301L as well as other *MAPT* mutations have been commonly used to model AD *in vivo* and *in vitro* despite their distinct physical properties (101). The genetically matched rT1 model overexpressing WT 0N4R human tau and rT2 model overexpressing P301L-tau also show differences in tau phosphorylation and stability at different developmental stages (102).

Evidence of involvement of Arc in AD pathology and the role of Arc in regulating learning and memory which are severely disrupted in AD raises the possibility of targeting Arc therapeutically to ameliorate some of these disruptions. Several drugs are known to alter Arc levels and function, including psychotropic drugs as well as other drugs that manipulate the proteasome and the autophagy-lysosome systems, most of which are well-studied and are already in use to treat other disorders (103). However, given the complexity of the role of Arc in AD, the desired effect of pharmacologically altering Arc remains unclear. More studies are needed to evaluate a role for Arc in AD that takes into consideration Arc in regulating AD pathology such as Aβ and the effect of tau pathology on regulating Arc. Notably, Arc is robustly induced with experiences that stimulate plasticity and is specifically targeted to stimulated synapses (104) and holistic approaches that are already in practice for AD management such as cognitive therapy have shown evidence of substantial benefits for AD patients, many of which can induce Arc in a non-pharmacological manner (105). Additionally, several drugs designed to reduce tau through immunotherapy are currently in clinical trials, which can possibly ameliorate the downstream effects of increased tau (106).

Our study identifies a new physiological role for tau in regulating Arc, a key regulator of synaptic plasticity (107). These findings carry implications for both tau and Arc. For tau, it suggests a new potential mechanism for its ability to regulate synaptic plasticity. While the role of Arc in regulating long-term potentiation has been brought into question (108), the role of Arc in regulating protein-synthesis-dependent forms of LTD is well-established, and Arc dysregulation could potentially be an underlying mechanism for the observed disruptions in LTD in *Tau* KO mice and AD animal models (108, 109, 110). For Arc, our findings further our understanding of its turnover and establish tau as a new Arc regulator. The inability of P301L-tau to modulate Arc highlights the importance of distinctions in downstream signaling mechanisms activated by different tau mutants involved in neurodegenerative disease.

## Experimental procedures

### Animals

All animal care and use were carried out following the National Institutes of Health Guidelines for the Use of Animals using approved protocols by the Georgia State University Institutional Animal Care and Use Committee. *Tau* KO mice and control WT C57BL/6J were obtained from The Jackson Laboratories (stocks #007251 and #000664) (111). The following primers were used to validate the genotype of *Tau* KO and C57BL/6J mice: mutant forward: 5′-GCCAGAGGCCACTTGTGTAG-3′, WT forward: 5′-AATGGAAGACCATGCTGGAG-3′, and Common: 5′- ATTCAACCCCCTCGAATTTT-3′ according to the protocol recommended by the Jackson Laboratory, with the *Tau* KO band at ∼170 bp, Heterozygote ∼170 bp and 269 bp, and WT at 269 bp. Animals used in fractionation experiments and primary hippocampal cultures are balanced for sex and littermates from heterozygous pairings.

### HEK293 cell line cultures and transfections

HEK293 cells were generously provided by Dr Jun Yin (Georgia State University). Cells were maintained in Dulbecco’s Modified Eagle Medium (Corning # 10013CV) with 10% fetal bovine serum and 1% penicillin-streptomycin (Thermo Fisher Scientific). Cells were transfected at 60 to 70% confluency with Lipofectamine 3000 (Thermo Fisher Scientific) according to the manufacturer instructions. To control for transfection efficiency, cells were plated at the same density between different conditions, and the plasmid pcDNA3.1 was added with single-transfections to counterbalance other conditions that had multiple DNAs that were transfected. Culture media was exchanged 4 h post-transfection to remove transfection material and cells were harvested 48 h later. For proteasome inhibition experiments, cells were treated with 10 μM MG-132 (Sigma # 474790) for 4 h before harvesting. MG-132 is an aldehyde peptide and a potent proteasome inhibitor that blocks the proteasome by forming a hemiacetal with the hydroxyl of the 20S active site threonines (95). For lysosome inhibition experiments, cells were treated with 50 μM leupeptin (Sigma # L2884) and 10 mM NH_4_Cl (Sigma # A9434) as described in (42) for 6 h before harvesting. For GSK3α/β inhibition, cultures were treated with 1 to 2 μM CHIR 98014 (Tocris #6695) as described in (45) for 4 h before harvesting.

Plasmids used for transfection include the following: pcDNA 3.1, pEGFP-C3 (Clontech), pCMV-tdTomato, pRK5-myc-Arc (generously provided by Dr Paul Worley, Johns Hopkins University), pRK5-myc-Arc5KR, pRK5-myc-Arc S170A/T175A, pRK5-myc-Arc T368A, pRK5-myc-Arc T380A, pRK5-myc-Arc K92Q, pRK5-myc-Arc ΔC-terminal, pRK5-myc-Arc ΔCC, pRK5-myc-Arc ΔEB (37), pRK5-EGFP, pRK5-EGFP-tau (Addgene #46904), and pRK5-EGFP-tau P301L (Addgene #46908).

### Cloning

pRK5-EGFP was generated using a PCR-based subcloning strategy. Briefly, EGFP was PCR amplified from the pRK5-EGFP-tau plasmid using primers with overhanging ends containing ClaI and SalI restriction sites with the addition of two STOP codons for the reverse primer. The pRK5-EGFP-tau plasmid and EGFP PCR product were digested with ClaI and SalI to remove EGFP-tau and then gel purified. EGFP was ligated into the cut plasmid using the Quick Ligation kit (New England Biolabs) per manufacturer instructions. Resulting colonies were then selected and the plasmids were purified using the QIAprep Spin Miniprep Kit (Qiagen). Purified plasmids were then screened for the correct insert size using ClaI and SalI restriction digest. Primers used for EGFP subcloning were as follows:

ClaI EGFP For.: 5′-GAAGAAATCGATGGTCGCCACCATGGTGAG-3′

SalI EGFP Rev.: 5′-GAAGAAGTCGACTTATTACTTGTACAGCTCGTCCATGC-3′

The QuickChange Site-directed mutagenesis procedure was used to generate the mutants pRK5-myc-Arc S170A/T175A, pRK5-myc-Arc T368A, pRK5-myc-Arc T380A, pRK5-myc-Arc K92Q from the pRK5-myc-Arc backbone. The primers used were as follows:

S170A/T175A For.: 5′-GGCTACGACTACACTGTTGCCCCCTATGCCATCGCCCCGCCACCTGCCGCAGGA-3′

S170A/T175A Rev.: 5′-TCCTGCGGCAGGTGGCGGGGCGATGGCATAGGGGGCAACAGTGTAGTCGTAGC-3′

T368A For.: 5′-GGCAGCTGAGCCTTCTGTCGCCCCTCTGCCCACAGAGGATG-3′

T368A Rev.: 5′-CATCCTCTGTGGGCAGAGGGGCGACAGAAGGCTCAGCTGCC-3′

K92Q For.: 5′-GGAAGAAGTCCATCCAGGCCTGTCTCTGC-3′

K92Q Rev.: 5′-GCAGAGACAGGCCTGGATGGACTTCTTCC-3′

The T380A mutant was generated using the overlap extension method as previously described in (112) with the flanking primers at the site of the mutation.

T380A 3′5′ flanking primer: 5′-GAAGTCGACCCCGGGAATGGAGCTGGA-3′

T380A 5′3′ flanking primer: 5′-GAAGGATCCTTACTTACTTAGCGGCCG 3′

Fwd.: 5′-GATGAGACTGGGGCACTCGCCCCTGCTCTTACCAGCGAG-3′

Rev.: 5′-CTCGCTGGTAAGAGCAGGGGCGAGTGCCCCAGTCTCATC-3′

BamHI and SalI restriction enzymes (New England Biolabs) were used to subclone the mutated fragment into the pRK5-myc-Arc backbone. Arc T380A was ligated into the BamHI and SalI cut pRK5-myc-Arc plasmid using the Quick Ligation kit per manufacturer instructions. Resulting colonies were then selected and the plasmids were purified using the QIAprep Spin Miniprep Kit (Qiagen). Purified plasmids were then screened for the correct insert size using BamHI and SalI restriction digest. All positive clones and point mutations were validated by Sanger sequencing.

### Western blotting

HEK293 cells were harvested and then cell pellets were lysed on ice in RIPA buffer (150 mM NaCl, 50 mM Tris–HCl, 1% v/v Nonidet P-40, 0.5% sodium deoxycholate, 0.1% SDS) with 1 mM DTT (Millipore) and protease inhibitors (0.1 mM PMSF (Calbiochem), 1 μM leupeptin (Millipore), 0.15 μM aprotinin (Millipore). Lysates were centrifuged at 13,000 rpm for 20 min at 4 °C to precipitate insoluble extracts. Protein concentrations were measured using the Pierce 660 assay (Thermo Fisher Scientific). To extract insoluble proteins, the precipitated pellet was resuspended in RIPA buffer then centrifuged at 13,000 rpm for 20 min at 4 °C, then resuspended in 70% formic acid and vortexed for 2 min. The samples were centrifuged at 13,000 rpm for 20 min at 4 °C; the pellet was discarded and 20 volumes of neutralization buffer (1 M Tris Base, 0.5 M Na_2_PO_4_ with protease and phosphatase inhibitors) were added to the supernatant.

Proteins were separated by SDS-PAGE and then transferred to nitrocellulose membrane at 4 °C (0.45 μm pore size, Bio-Rad). Membranes were blocked overnight at 4 °C in Intercept tris-buffered saline (TBS) blocking buffer (LI-COR) and then incubated in primary antibodies in 1:1 blocking buffer to 1% Tween-20 (Acros) in TBS (TBST) with 0.02% NaN_3_ overnight at 4 °C. Membranes were washed 3 times with double distilled water for 5 min, and secondary antibodies in 1:1 blocking buffer to TBST 0.1% SDS (Bio-Rad) were added to the membranes for 1 h at room temperature, then washed 2 times with TBST and 1 time with double distilled water for 5 min.

Primary antibodies used are as follows: mouse anti-myc (Santa Cruz #sc-40) at 1:1000, rabbit anti-GFP (Novus Biologicals # NB600-308) at 1:1000, mouse anti-β Actin (Genetex #GTX629630) at 1:3000, mouse anti-Tau-1 (Millipore Sigma #MAB3420MI) at 1:1000, mouse anti-Tubulin [GT114] (Genetex # GTX628802) at 1:1000, mouse anti-Ubiquitin [P4D1] (Santa Cruz #sc-8017) at 1:500, rabbit anti-LC3 (Novus Biologicals #NB100-2220) at 1:500, rabbit anti-p62 (Proteintech #18420-1-AP) at 1:1000.

Secondary antibodies used are as follows: IRDye goat anti-mouse 680RD (LI-COR #926-68070) at 1:20,000; IRDye donkey anti-rabbit 680RD (LI-COR # 925-68073) at 1:20,000; IRDye donkey anti-mouse 800CW (LI-COR #926-32212) at 1:15,000; IRDye goat anti-rabbit 800CW (LI-COR # 926-32211) at 1:15,000.

Western blot membranes were scanned using the LI-COR Odyssey CLx scanner (low scan quality, 163 μm scan resolution, auto channel intensities). Images were analyzed using ImageJ software (imagej.net/ij/download.html) (NIH) with the Gel Analysis tool or Image Studio Lite software (Li-COR Biosciences). To adjust high background, the Subtract Background tool in FIJI was used on the whole channel with a rolling ball radius of 20 to 50 pixels.

### In-cell western assay

One day before transfection, HEK293 cells were plated on poly-d-lysine-coated (Millipore #P7280) (0.01 mg/ml) 96-well plates (Thermo Fisher Scientific#269787) at an identical density. Cells were transfected at 60 to 70% confluency with Lipofectamine 2000 (Invitrogen) according to the manufacturer instructions. Cells were fixed 24 h later and processed similarly to our previous work (61, 62) with the following modifications: briefly, the media was removed and replaced with room temperature 4% Sucrose/4% paraformaldehyde and incubated at room temperature for 20 min. Cells were then washed twice with PBS containing Mg^2+^/Ca^2+^ (Corning) and permeabilized at room temperature for 15 min in 0.1% Triton X-100 (Thermo Fisher Scientific) in PBS containing Mg^2+^/Ca^2+^. Intercept TBS blocking buffer (LI-COR) was added to the cells and incubated at room temperature for 2 h. Following the blocking step, an anti-GFP and anti-Myc antibody solution (1:1000 Rb-GFP (Clontech), 1:500 ms-Myc (Santa Cruz) in a 1:1 solution of PBS containing Mg^2+^/Ca^2+^ and LI-COR TBS blocking buffer) was added to the cells and incubated overnight at 4 °C. Cells were washed 3 times with PBS containing Mg^2+^/Ca^2+^ and then a secondary antibody solution (1:1500 IRDye donkey anti-mouse 800CW, 1:1500 IRDye donkey anti-rabbit 680RD in a 1:1 solution of PBS containing Mg^2+^/Ca^2+^ and LI-COR TBS blocking buffer) was added to the cells and incubated for 1 h at room temperature in the dark. Cells were then washed 5 times with PBS containing Mg^2+^/Ca^2^. The 96-well plate was scanned using the LI-COR Odyssey CLx scanner (auto scan feature, resolution of 84 μm, medium quality, and 3 mm focus offset). Images were analyzed as described previously (62).

### Co-immunoprecipitation

Transfected cells were lysed in IP buffer (20 mM Tris–HCl, 3 mM EDTA, 3 mM EGTA, 150 mM NaCl, 1% Triton X-100, pH 7.4) with 1 mM DTT, protease and phosphatase inhibitors (0.1 mM PMSF, 1 μM leupeptin, 0.15 μM aprotinin, and 1:2000 Halt phosphatase inhibitor cocktail; Thermo Fisher Scientific #78420). Lysates were centrifuged at 13,000 rpm for 20 min at 4 °C to precipitate insoluble proteins. Protein concentration was determined using the Pierce 660 assay (Thermo Fisher Scientific). One milligram of protein was used for each condition and brought up to a total volume of 1 ml in IP buffer. Beads (Protein A/G PLUS-Agarose; Santa Cruz #sc-2003) were pre-equilibrated in IP buffer with inhibitors. Protein samples were incubated with 2.5 μg/sample of the primary antibody (goat anti-myc (Bethyl #A190-104A) or mouse anti-myc (Santa Cruz #SC-40)) and left to tumble for 1 h at 4 °C, then an equal volume of beads suspension was added per sample and left to tumble overnight. Samples were centrifuged for 45 s at 13,000 rpm to pellet the beads. The supernatant was discarded, and beads were washed 3 times for 5 min with IP buffer before adding 2× SDS sample buffer (4% SDS, 20% glycerol, 0.2% bromophenol blue, 3% DTT, 0.1 M Tris–HCl, 1:1000 β-mercaptoethanol, pH 6.8) and heating to 45 °C for 5 min. Proteins were then separated using SDS-PAGE as described above.

For the ubiquitination assay, the same protocol was followed except that RIPA buffer was used instead of IP buffer.

### Tissue fractionation

Dissected hippocampi from 3-month-old WT and *Tau* KO mice of mixed sex were homogenized in 10 volumes of Hepes-buffered sucrose (0.32 M sucrose, 4 mM Hepes, pH 7.4) with 1 mM DTT and protease inhibitors (0.1 mM PMSF, 1 μM leupeptin, 0.15 μM aprotinin). Tissue homogenate was spun at 800*g* for 15 min at 4 °C to precipitate the nuclear fraction (P1). The resulting supernatant (S1) was spun at 10,000*g* for 15 min to yield the crude synaptosomal pellet (P2). P2 was washed by resuspending in 10 volumes of Hepes-buffered sucrose and re-spinning at 10,000*g* for 15 min. P2 was lysed by hypoosmotic shock in nine volumes of ice cold water with inhibitors and then rapidly adjusted to 4 mM Hepes using 1 M Hepes, pH 7.4, then left to tumble at 4 °C for 30 min. Samples were then centrifuged at 25,000*g* for 20 min to yield the supernatant S3 (synaptosomal vesicle fraction) and the pellet P3 (synaptosomal membrane fraction). P3 was resuspended in Hepes-buffered sucrose. Quantification of protein concentrations was done using the Pierce assay and 7 μg of protein/fraction was used in Western blot analysis.

### Primary hippocampal neuronal cultures

Primary hippocampal neurons of mixed sex were isolated from P0-1 mice as previously described (41) and cultured on poly-d-lysine-coated coverslips (0.1 mg/ml) in 24-well plates at a density of 75,000 cells/well. Cultures were maintained in neuronal feeding media: Neurobasal media (Gibco) containing 1% GlutaMAX (Gibco), 2% B-27 (Gibco), 4.8 μg/ml 5-Fluoro-2′-deoxyuridine (Sigma), and 0.2 μg/ml Gentamicin (Sigma). On DIV 6, half the media was replaced with prewarmed fresh neuronal feeding media. Cultures were transfected with Lipofectamine 2000 on DIV 9 to 12 as described in (37) with an equal amount of cDNA transfected into all conditions. TdTomato or GFP was used as a cell fill to identify neuron morphology as described in (102, 113). For proteasome inhibition experiments, cultures were treated with 0.5 μM tetrodotoxin citrate (TTX; Tocris #1069) and 10 μM MG-132 for 4 h before they were fixed.

### Immunocytochemistry

Forty eight hours after transfection, cultures were treated with TTX and then fixed for 20 min at 4 °C with 4% Sucrose/4% paraformaldehyde. Neurons were permeabilized with 0.2% Saponin for 15 min then blocked in 10% normal horse serum (NHS) in PBS for 1 h at 37 °C. Permeabilization was skipped for surface GluA1 labeling. Neurons were then incubated overnight in primary antibody in 3% NHS, then washed and incubated in secondary antibody at 1:1000 and DAPI at 1:2000 in 3% NHS for 1 h in the dark at room temperature. Coverslips were washed with PBS and then mounted onto slides with Fluorogel (Electron Microscopy Sciences).

Primary antibodies used were as follows: rabbit anti-Arc (synaptic systems #156003) at 1:500, mouse anti-GluA1 (Millipore MAB2263) at 1:150. Secondary Antibodies used: Donkey anti-rabbit AlexaFluor 647 (ThermoFisher #A31573), Goat anti-mouse AlexaFluor 647 (ThermoFisher #A21240).

### Image acquisition and analysis

For primary hippocampal neurons, coverslips were imaged on a Zeiss LSM 700 confocal microscope under 40× (Arc experiments NA 1.4, Zeiss #420762-9900) or 63× (NA 1.4, Zeiss #420782-9900-000, GluA1 experiments) immersion lens. 12-step raw z-stack images were acquired with step size 0.42 μm. Acquisition parameters were kept constant between different conditions within the same experiment and samples were interleaved during imaging. Images were analyzed using ImageJ software (NIH). Regions of interest were manually outlined (guided by the neuron morphology visualized by the tdTomato or GFP cell fill), and integrated density values were quantified for Arc and GluA1 in the initial 20 μm of apical dendrites. Dendritic spines were quantified manually on the GluA1-labeled neurons imaged at 63× on the z-stacks guided by neuron morphology visualized by tdTomato cell fill in ImageJ. Protrusions from a maximum of 100 μm length of apical dendrites and their main branch less than or equal to 3 μm and with an expanded head were counted as spines and the number of spines per dendrite was normalized to the length of the dendrite.

### Re-analysis of the AD brain proteome

Proteomic and protein-specific correlation data for various disease parameters of Arc such as MMSE score, Braak stage, CERAD, and Aβ plaque levels were collected from a publicly available database associated with publication in (60).

### Experimental design and statistical analysis

All experiments follow a between-subjects design. Statistical analysis was conducted using GraphPad prism as described in the text for each experiment. Nonparametric tests are used when the criteria for using parametric tests are not met. Data is represented as mean ± SEM with statistical significance set at 95%.

## Data availability

All data and materials are available upon request from the lead contact author.

## Supporting information

This article contains supporting information.

## Conflict of interest

The authors declare that they have no conflicts of interests with the contents of this article.

## Figures and Tables

**Supporting Figure S1:**