# Supplementary material for: Tau regulates Arc stability in neuronal dendrites via a proteasome-sensitive but ubiquitin-independent pathway
Source: J Biol Chem. 2024 Mar 27;300(5):107237. doi: 10.1016/j.jbc.2024.107237 (PMC11061231; doi:10.1016/j.jbc.2024.107237)
Supplement: Supporting Figure legends [file mmc1.docx]

**Supplemental Figure 1: Validation of *Tau* KO mice and subcellular fractionation from hippocampus**

***A,*** Example of successful genotyping of *Tau* KO mice.

***B,*** Representative western blot showing the absence of tau protein in *Tau* KO mice. Unpaired t-test t = 12.22, df = 12, p < 0.0001

***C,*** Representative western blots showing Arc, GluA1, PSD-95, and Actin in total hippocampal (Hp) lysates. Hippocampi were harvested from 3-month-old WT and *Tau* KO littermates from both sexes. Arc was significantly higher in total Hp lysates. Unpaired t-test, t = 2.42, df = 12, p = 0.032.

***D*,** *Left,* Representative western blots showing GluA1, PSD-95, and Actin in total hippocampal lysates and subcellular fractions. Quantification of PSD-95 and GluA1 across all probed subcellular fractions. *Right,* Values are normalized to actin levels within the same fraction. One-way ANOVA for PSD-95, F (4, 29) = 1.988, p = 0.1228. Kruskal-Wallis for GluA1 p = 0.002. n = 6 mice

**Supplemental Figure 2: Activity-dependent reduction of Arc in dendrites is tau-dependent**

***A,*** Representative images of primary hippocampal neurons from WT and *Tau* KO littermates transfected at DIV 9 with GFP to outline neuron morphology, treated with TTX at DIV10, and fixed at DIV11. Scale bar = 20 μm. Scale bar in selected dendrites = 5 μm.

***B,*** Quantification of Arc in dendrites showing a lack of TTX-induced Arc reduction in *Tau* KO neurons. t-test, t = 2.517, df = 29, p = 0.0176; n = 15-16 neurons from 3 independent biological replicates.

***C,*** Quantification of Arc in soma showing a lack of TTX-induced Arc reduction in both genotypes. t-test, t = 0.677, df = 29, p = 0.504. n = 15-16 neurons from 3 independent biological replicates.

**Supplemental Figure 3: Overexpression of GFP-tau or GFP-P301L tau does not alter dendritic spine densities.**

Quantification of dendritic spines on apical dendrites per 1 μm. One-way ANOVA, p = 0.857. Scale bar = 5 μm

**Supplemental Figure 4: P301L-tau overexpression and blocking known Arc ubiquitination sites does not decrease myc-Arc**

***A,*** Representative western blots showing myc-Arc with increasing concentrations of GFP- P301L tau in HEK293 cells (0, 0.25, 0.5, 0.75, 1 and 1.5 μg). pcDNA3.1 was used as a DNA filler to keep the amount of transfected DNA between titration conditions identical. Actin was used as a loading control. *Bottom*, Quantification of myc-Arc normalized to actin showing no significant differences in Arc with increasing P301L tau. One-way ANOVA, F (5,18) = 0.3018, p = 0.3. n= 4.

***B,*** Schematic showing the structure of Arc, highlighting the coiled-coil (CC) domain and the endophilin-binding (EB) domain on the N-terminus, and the N- and C-lobe on the C-terminus. The locations of the lysines (K55,136, 268, 269, and 293) targeted for ubiquitination by RNF216 and UBE3A are shown.

***C,*** *Left,* Representative western blots showing myc-Arc5KR (K55,136, 268, 269 and 293 mutated to Arginine) with increasing concentrations of GFP-tau in HEK293 cells (0, 0.25, 0.5, 0.75, 1 and 1.5 μg). pcDNA3.1 was used as a DNA filler to keep the amount of transfected DNA between titration conditions identical. Actin was used as a loading control. *Right*, Quantification of myc-Arc5KR levels normalized to actin showing a significant decrease in Arc with increasing tau. One-way ANOVA F = (5,18) = 6.4, p = 0.0014. n = 4.

**Supplemental Figure 5: Tau does not promote lysosomal degradation of Arc**

***A,*** Representative western blots showing myc-Arc expressed alone or with GFP-tau. Cells were treated with Vehicle (water) or a combination of leupeptin (50 μM) and ammonium (10 mM) chloride for 6 hr to block lysosome degradation. Actin was used as a loading control. Blots showing markers for lysosome inhibition, LC3-II, and p62/SQSTM1 with total protein stain used as a loading control.

***B,*** Quantification of LC3-II and p62/SQSTM1 showing a significant increase of LC3-II (unpaired t-test, t = 12.52, df = 22, ****p < 0.0001) and p62/SQSTM1 (unpaired t-test, t = 10.54, df = 22, ****p < 0.0001) in cells from all conditions treated with inhibitors. n = 12.

***C,*** Quantification of myc-Arc showing a significant decrease with co-expression of GFP-tau in Vehicle and following lysosome inhibition. t-test for vehicle control, t = 10.6, df = 10, ****p < 0.0001; unpaired t-test for inhibitors, t = 5.585, df = 10, p = 0.0002. n = 6.

***D,*** Representative western blots showing RIPA-soluble and insoluble fractions of myc-Arc expressed alone or with GFP-tau.
