# Supplementary figures and images for: Tau regulates Arc stability in neuronal dendrites via a proteasome-sensitive but ubiquitin-independent pathway
Source: J Biol Chem. 2024 Mar 27;300(5):107237. doi: 10.1016/j.jbc.2024.107237 (PMC11061231; doi:10.1016/j.jbc.2024.107237)

A

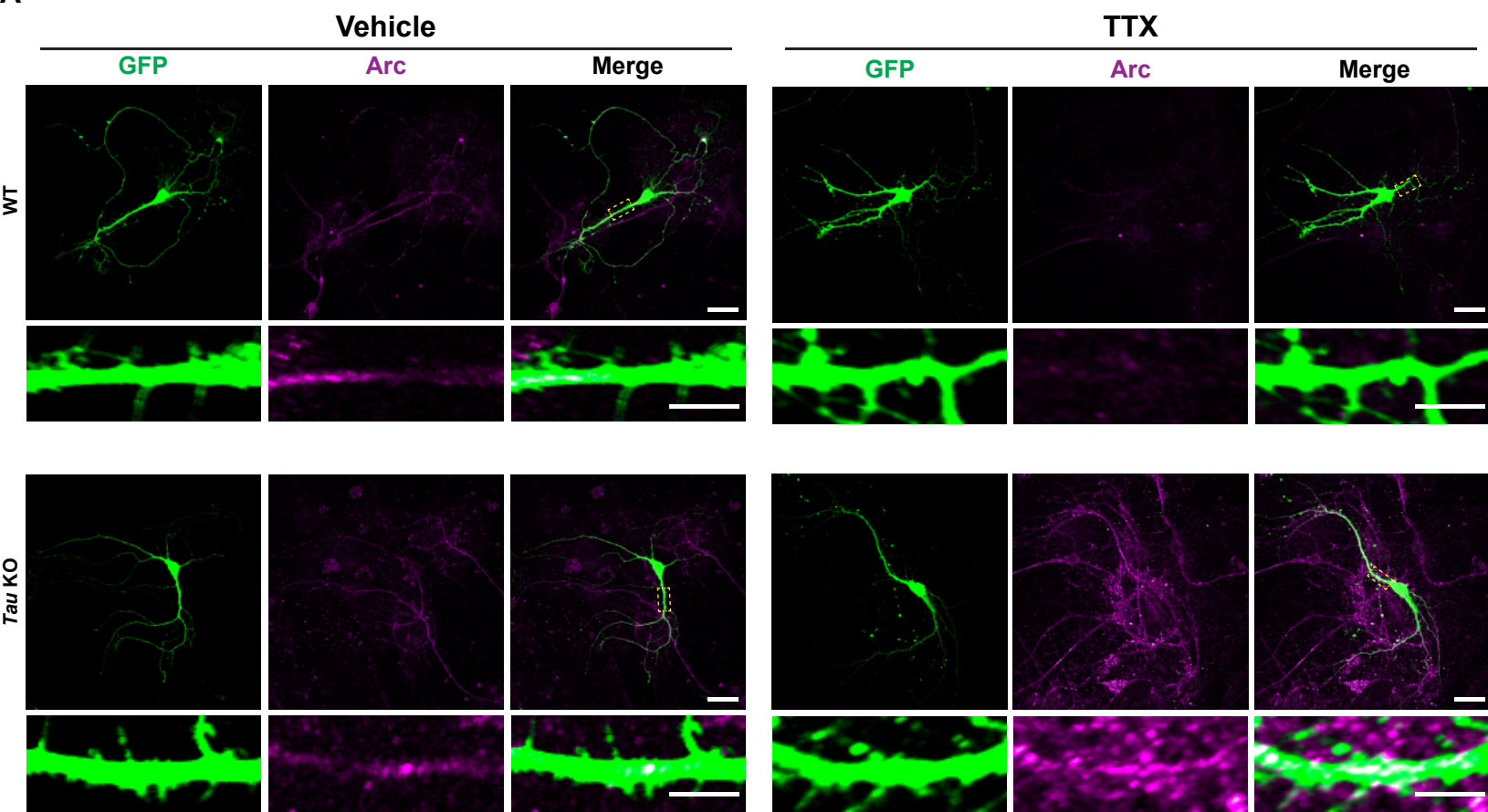

B

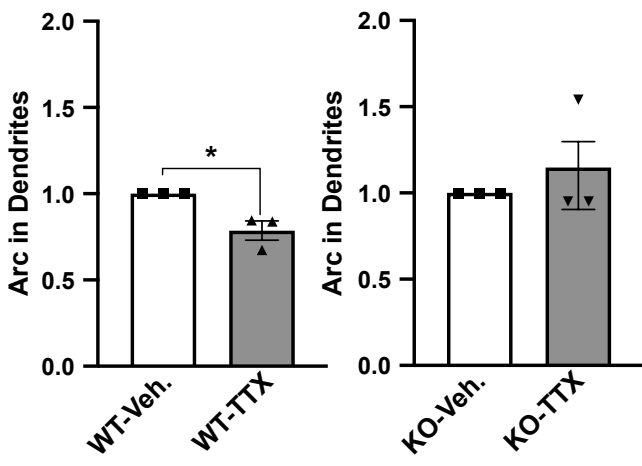

C

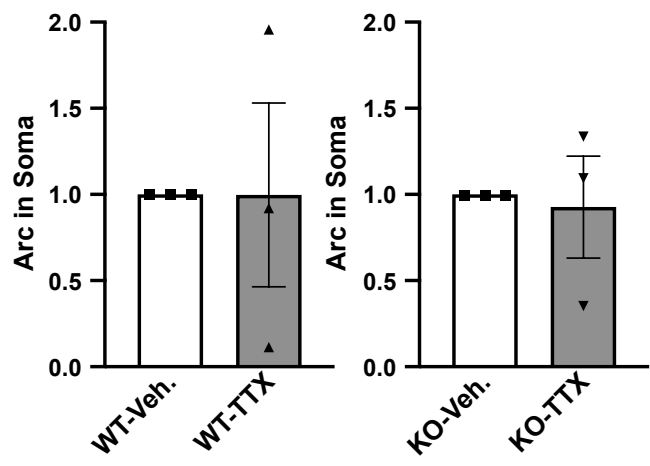

Supplement: Supporting Figure S2 [file mmc2.pdf]

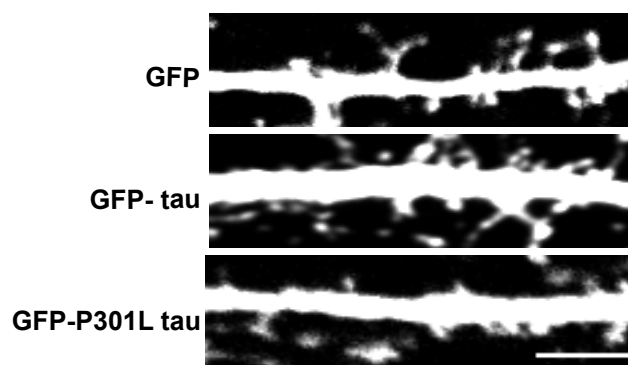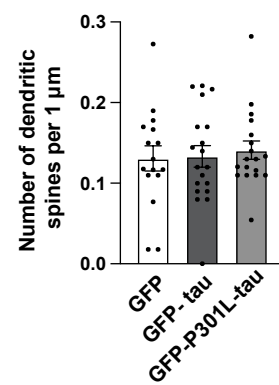

Supplement: Supporting Figure S3 [file mmc3.pdf]

**A**

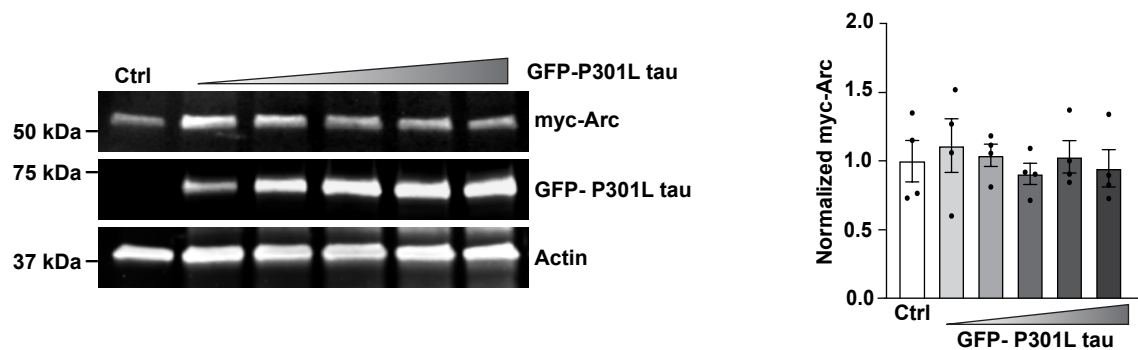

**B**

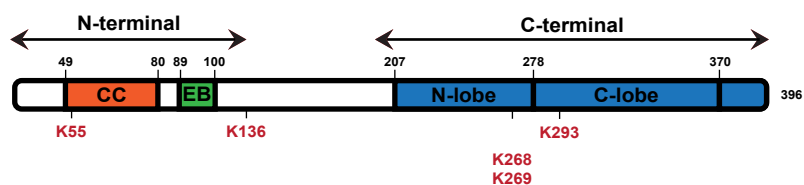

**C**

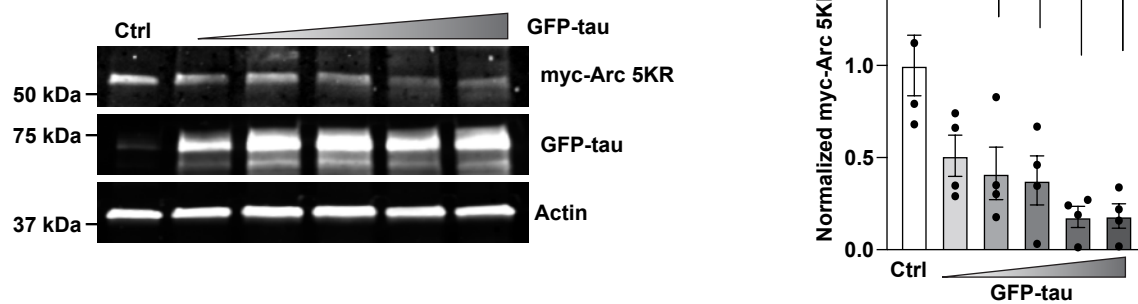

Supplement: Supporting Figure S4 [file mmc4.pdf]

**A**

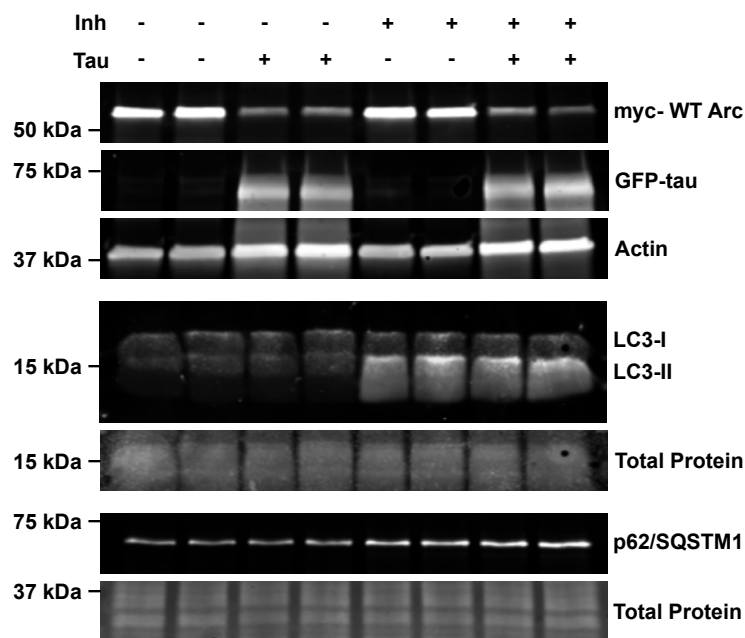

**B**

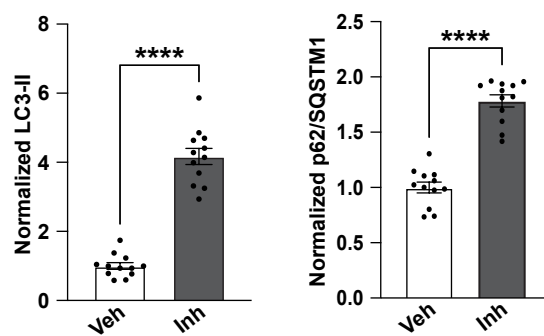

**C**

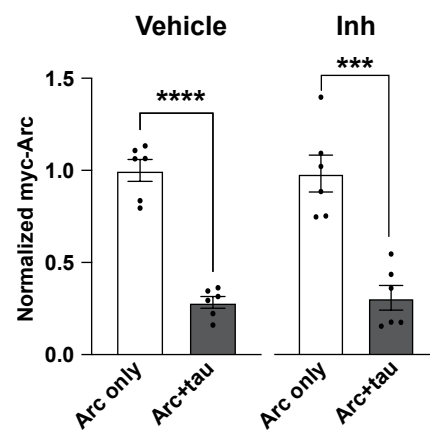

**D**

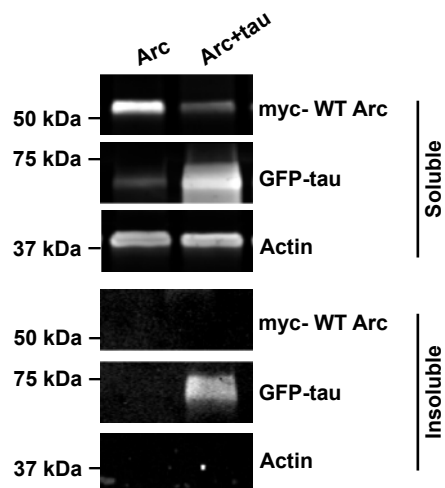

Supplement: Supporting Figure S5 [file mmc5.pdf]
